# A novel high-throughput method for kinetic characterisation of anaerobic bioproduction strains, applied to *Clostridium kluyveri*

**DOI:** 10.1038/s41598-018-27594-9

**Published:** 2018-06-27

**Authors:** Pieter Candry, Timothy Van Daele, Kyrina Denis, Youri Amerlinck, Stephen J. Andersen, Ramon Ganigué, Jan B. A. Arends, Ingmar Nopens, Korneel Rabaey

**Affiliations:** 1 Center for Microbial Ecology and Technology (CMET), Faculty of Bioscience Engineering, Ghent University, Coupure Links 653, 9000 Gent, Belgium; 2 BIOMATH, Department of Mathematical Modelling, Statistics and Bioinformatics, Faculty of Bioscience Engineering, Ghent University, Coupure Links 653, 9000 Gent, Belgium

## Abstract

Hexanoic acid (HA), also called caproic acid, can be used as an antimicrobial agent and as a precursor to various chemicals, such as fuels, solvents and fragrances. HA can be produced from ethanol and acetate by the mesophilic anaerobic bacterium *Clostridium kluyveri*, via two successive elongation steps over butyrate. A high-throughput anaerobic growth curve technique was coupled to a data analysis framework to assess growth kinetics for a range of substrate and product concentrations. Using this method, growth rates and several kinetic parameters were determined for *C*. *kluyveri*. A maximum growth rate (µ_max_) of 0.24 ± 0.01 h^−1^ was found, with a half-saturation index for acetic acid (K_S,AA_) of 3.8 ± 0.9 mM. Inhibition by butyric acid occurred at of 124.7 ± 5.7 mM (K_I,BA_), while the final product, HA, linearly inhibited growth with complete inhibition above 91.3 ± 10.8 mM (K_HA_ of 10.9*10^−3^ ± 1.3*10^−3^ mM^−1^) at pH = 7, indicating that the hexanoate anion also exerts toxicity. These parameters were used to create a dynamic mass-balance model for bioproduction of HA. By coupling data collection and analysis to this modelling framework, we have produced a powerful tool to assess the kinetics of anaerobic micro-organisms, demonstrated here with *C*. *kluyveri*, in order further explore the potential of micro-organisms for chemicals production.

## Introduction

**Figure 1:**
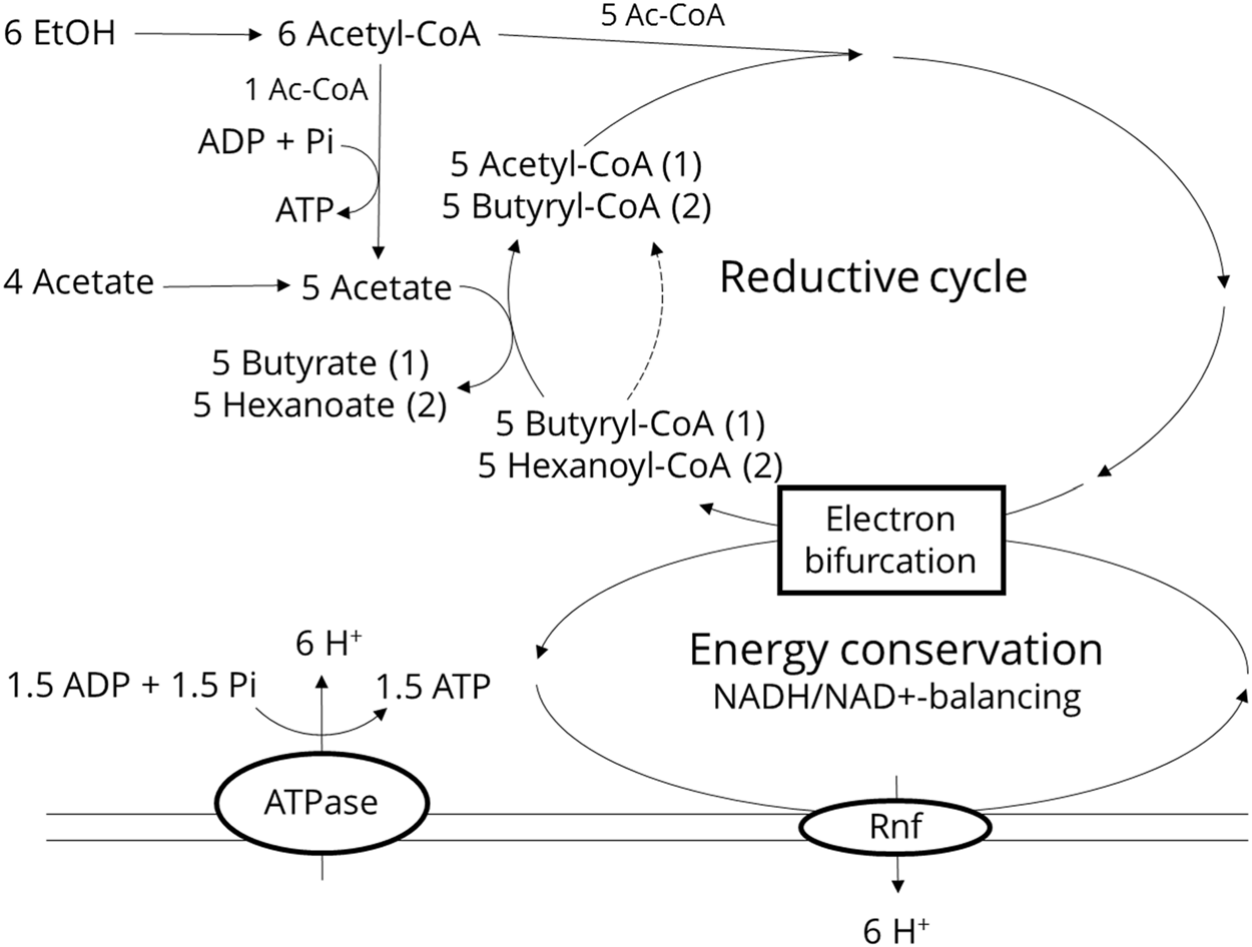
Schematic representation of reverse beta-oxidation cycle, adapted from Angenent *et al*.^1^. Briefly, the metabolism first converts EtOH to acetyl-CoA, part of which (one molecule in six) is oxidised to acetate for ATP-generation. The remaining acetyl-CoA is combined with acetyl-CoA cycled in the reverse β-oxidation cycle, producing butyryl-CoA. This butyryl-CoA can either be converted to butyrate, or immediately be cycled again (dashed arrow), yielding hexanoyl-CoA, which in turn can be converted to hexanoate. Simultaneously 6 protons are pumped across the cell membrane per cycle, required to balance the NADH/NAD^+^-pool over the entire metabolism. This process generates a proton motive force, which allows to recover more of the energy generated in the cycle (in total 2.5 ATP per cycle).

Microbial production of chemicals, fuels and materials from low-value solid, liquid and gaseous streams is gaining momentum. Hexanoic acid (HA) is a six-carbon medium chain carboxylic acid (MCCA) that is conventionally sourced from plant oils and animal fats^1^, but is also a microbial product that can be produced from various organic wastes. HA is used as an antimicrobial agent in pig feed, and can also be converted to fragrances, flavours, and jet fuels^1^. Microbial HA production was first described in *Clostridium kluyveri*, after isolation by Barker and Taha^2^. This bacterium converts ethanol (EtOH) and acetic acid (AA) to HA through the reverse β-oxidation pathway, and has been thoroughly described in literature^1,3–6^. For the scope of this study, the metabolism (Fig. 1) can be simplified to a two-step reaction process (reactions (1) and (2)) in which acetate is first converted to butyrate and subsequently to hexanoate, with EtOH acting as the electron donor driving both reactions.

1 $$6\,{\rm{EtOH}}+4\,{{\rm{acetate}}}^{-}\to \,{{\rm{butyrate}}}^{-}+{{\rm{H}}}^{+}+2\,{{\rm{H}}}_{2}+4\,{{\rm{H}}}_{2}{\rm{O}}$$

2 $$6\,{\rm{EtOH}}+5\,{{\rm{butyrate}}}^{-}\to {{\rm{hexanoate}}}^{-}+{{\rm{acetate}}}^{-}+{{\rm{H}}}^{+}+2{{\rm{H}}}_{2}+4{{\rm{H}}}_{2}{\rm{O}}$$

In contrast to the extensive knowledge of *C*. *kluyveri*’s metabolism, there is little data available for key growth parameters. Thauer *et al*.^4^, for instance, determined a biomass yield for EtOH (Y_EtOH_) of 1.5 mg of cell dry weight (CDW) per mM of EtOH consumed in batch conditions, while Kenealy & Waselefsky^7^, obtained Y_EtOH_ between 1.73 and 3.14 mg CDW.mM EtOH^−1^ in a series of chemostat experiments, depending on the conditions applied. Kenealy & Waselefsky originally claimed that no half-saturation indices could be determined with the available data. A biokinetic model was recently constructed and several kinetic parameters were estimated by fitting the constructed model to the original chemostat data^7,8^, which included half-saturation indices for AA (K_S,AA_ of 3.5 mM), butyric acid (BA, K_S,BA_ of 3.5 mM) and EtOH (K_S,EtOH_ of 11.8 mM). The inhibition constant for HA (7.5 mM undissociated HA) was extrapolated from studies on mixed cultures operated at pH 5.5, assuming only the undissociated form of HA to be toxic. The limited data on the kinetic properties of *C*. *kluyveri* – or microbial MCCA-production processes in general – requires expansion to obtain detailed information on growth rates, substrate affinity, substrate inhibition and product inhibition. This knowledge is of value to enable microbial production of HA, and ideally towards more sustainable carbon sources for HA.

It is essential to understand the underlying kinetics of a biocatalyst to engineer and operate a biological production system. This is particularly important when this kinetic knowledge can be translated into a process model that allows (in-silico) optimisation of reactor design for the process under consideration^9,10^. However, many modelling methods either require a (semi)-continuous reactor system at different operational conditions, or an extensive array of batch tests to estimate and validate the parameters^9^. Reactor-based characterisation can slow the design and optimisation of microbial biocatalyst production processes up front, and do not allow straight-forward monitoring of the kinetics. In addition, working with strictly anaerobic, axenic cultures is labour-intensive and often difficult to manage in large batch experiments.

In this study, we have further developed a high-throughput method to track growth of micro-organisms under a wide range of conditions^11^ which can reduce the effort and time investment to establish and monitor the kinetics of microbial bio-production systems. This technique was applied to *C*. *kluyveri* as a model biocatalyst.

## Results

### Product profiles in 96-well plate vs. Balch tubes deviate only under specific conditions

The validity of the novel, 96-well plate (96-WP) based experimental method was demonstrated by comparing product output of the pooled replicates in a 96-WP with that of a culture grown by a traditional method i.e. in a Balch tube. The cultivation methods generally give similar results, indicating that the 96-WP methodology is a valid means to assess kinetics, but under certain conditions deviations can be observed between the two. For the sake of clarity, the abbreviations AA, BA and HA from here on refer to the total pool of carboxylic acids and carboxylate anions, except where explicitly mentioned otherwise.

**Figure 2:**
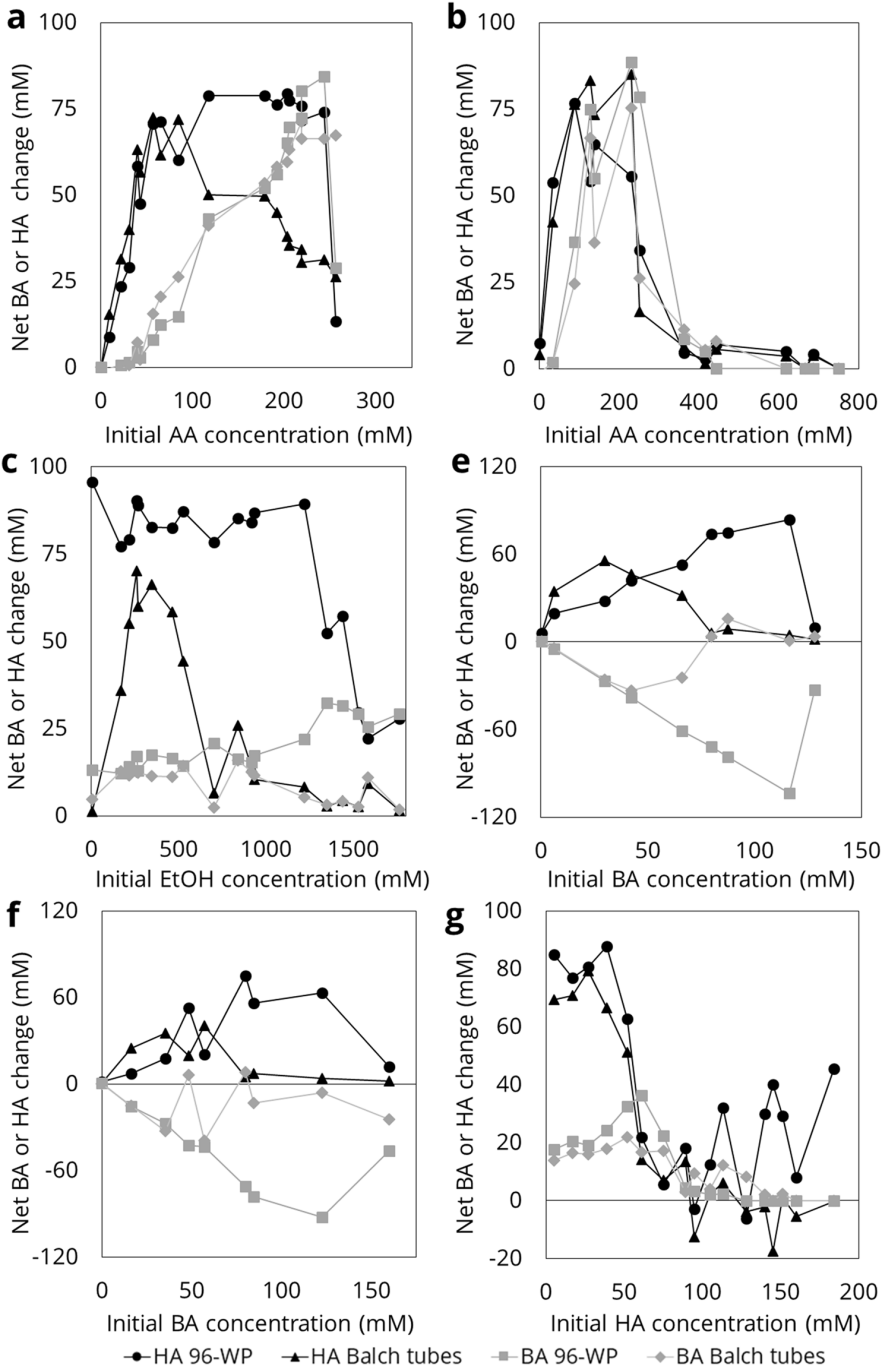
Comparison of butyric acid (BA) and hexanoic acid (HA) production or consumption in Balch tubes vs. 96-WP at the end of all experiments (90–100 h). HA change in 96-well plates (96-WP) represented by black circles (●), BA in 96-WP by grey squares (). HA change in Balch tubes is represented by black triangles (▲), BA in Balch tubes by grey diamonds () at the end of each experiment. Label for each subfigure refers to experiment number (Table 1). Experiment D did not result in any observable growth due to a lack of acetic acid (AA) in the medium (Supplementary Information S.2.5.).

At high initial substrate concentrations, *C*. *kluyveri* produces more BA and HA in a 96-WP compared to growth in Balch tubes, while at low initial electron acceptor concentrations, production is lower in 96-WP than in Balch tubes (Fig. 2a,b,e,f). If an initial AA concentration of over 100 mM was imposed (Exp A, B; EtOH fixed at 343 mM), or of BA above 60 mM (Exp E, F; EtOH fixed at 343 mM) supplemented with AA, HA-production is consistently higher in the 96-WP. The HA-toxicity experiment (Experiment G, Fig. 2f) also shows that *C*. *kluyveri* produces more HA in 96-WP when initial HA is below the toxicity limit. At higher initial HA-concentrations, no growth was observed in either 96-WP or Balch tubes, implying other processes, such as evaporation, could explain the observed net changes in HA-concentration. The experiment examining inhibition of EtOH (Experiment C, Fig. 2c) was not considered further, due to issues with evaporation and transfer of EtOH from wells with high concentrations of EtOH to wells with low concentrations of EtOH (Supplementary Information S.1.3., S.2.1., S.2.2.).

### Determination of growth parameters

#### Yield (Y)

Biomass productivity was determined in bottles, resulting in a Y_EtOH_ of 2.75 ± 0.78 mg VSS.mM EtOH^−1^. Yield was expressed as a function of the electron donor, ethanol, to be comparable with previous reports^4,7^. Kenealy & Waselefsky^7^ reported a Y_EtOH_ of 1.73–3.14 mg CDW.mM EtOH^−1^, in chemostats at different conditions in steady state (n >  = 6), while Thauer *et al*.^4^ reported a Y_EtOH_ of 1.5 mg CDW.mM EtOH^−1^ (n > = 5). The causes for these deviations could be found in two slight differences in the methods used. Thauer *et al*.^4^ used a growth medium without any yeast extract, instead only supplementing the necessary vitamins (biotin and p-aminobenzoic acid), while in both this work and the study by Kenealy & Waselefsky^7^ yeast extract was used to cultivate *C*. *kluyveri*. The use of yeast extract might increase the biomass yield of *C*. *kluyveri* although no data is available to confirm this. Secondly, Kenealy & Waselefsky used optical density (OD) to quantify biomass concentrations^7^, which is prone to shifts over time for *C*. *kluyveri* (Supplementary Information S.2.4.). Therefore, a direct determination of biomass is preferred, for example via VSS.

**Figure 3:**
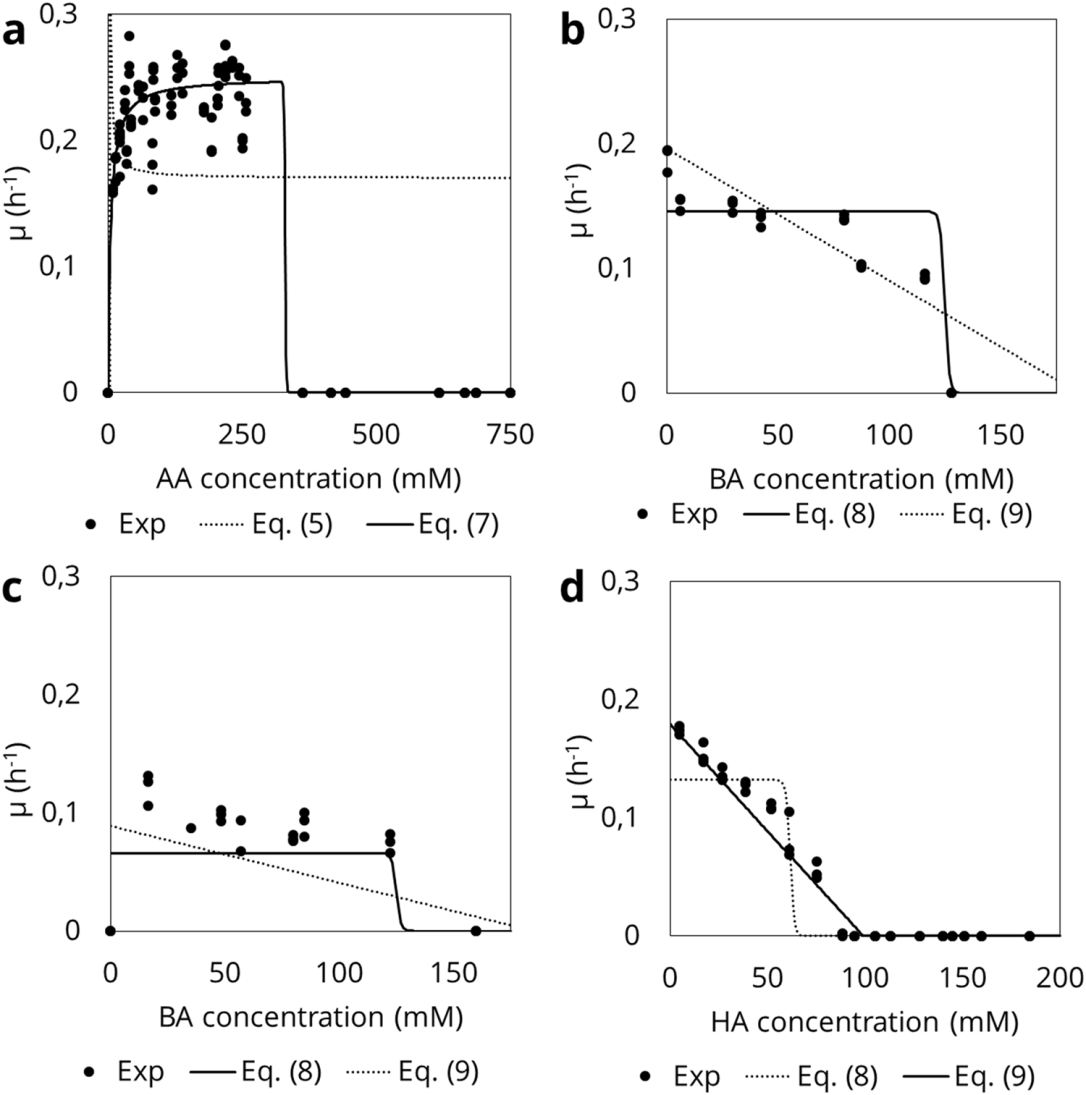
Comparison of proposed model types (Table 3) for each organic acid involved in the metabolism of *C*. *kluyveri*. Full circles (●) represent experimental data, lines indicate calibration of the proposed models for that compound, full lines are the best fit, dashed lines show the next best fit. Figure A shows the case for acetic acid (AA) with data from experiments A and B, Haldane model (Eq. (6)) was not included as no proper fit could be achieved. Figures B and C show data from experiments E and F and model selection for butyric acid (BA), calibrating the proposed models with data from both experiments, incorporating the effect of AA into the data. Figure D shows the case for hexanoic acid (HA) and proposed models, calibrated using data from experiment G.

#### Kinetic model

Model selection per compound: The growth kinetic response of *C*. *kluyveri* to different concentrations of carboxylic acids should be explained by the most suitable model for each acid tested (Fig. 3, Supplementary Information S.2.6.). The growth rate data revelead that µ first increases and then remains constant at approx. 0.25 h^−1^ for increasing AA concentrations. A critical initial concentration of AA is reached (between 260 and 360 mM AA) when the initial concentration of AA in increase further, above which no growth was observed (Fig. 3a). Notably, no good fit was achieved by the Haldane function, which predicts that µ asymptotically approaches 0 at high substrate concentrations. AA toxicity can be described by a Monod model coupled with a toxicity limit term. The toxicity limit (K_I,AA_) was set at 330 mM AA to correspond with the observed drop in µ between 260 mM and 360 mM.

The case for BA is more complex because low concentrations of AA are initially needed for *C*. *kluyveri* to start growing and consuming BA, which has been reported in literature^3^ and supported by the inconsistent growth observed in Experiment D (Supplementary Information S.2.5.). As a consequence, it was not possible to decouple growth on AA and on BA. In the model selection, the effect of AA was incorporated by adding a Monod-term for AA, using the K_S_-value obtained for the Monod model coupled with a toxicity limit term (4.7 mM AA, Supplementary Information S.2.6.). No half-saturation index (K_s, BA_) for BA was incorporated in the parameter estimation due to the lack of data at low BA-concentrations, and the need for AA to initialize growth. At higher BA-concentrations, a toxicity limit model gives the best performance due to the sudden decrease in µ at high concentrations (Fig. 3b,c). Finally, the response of *C*. *kluyveri* to HA was best described by a linear inhibition model (Fig. 3d). Monod kinetics for growth of *C*. *kluyveri* on HA were not incorporated, nor were any toxicity effects by octanoic acid taken up in the model as only traces (<2 mM) of octanoic acid (C8 MCCA) were detected.

**Figure 4:**
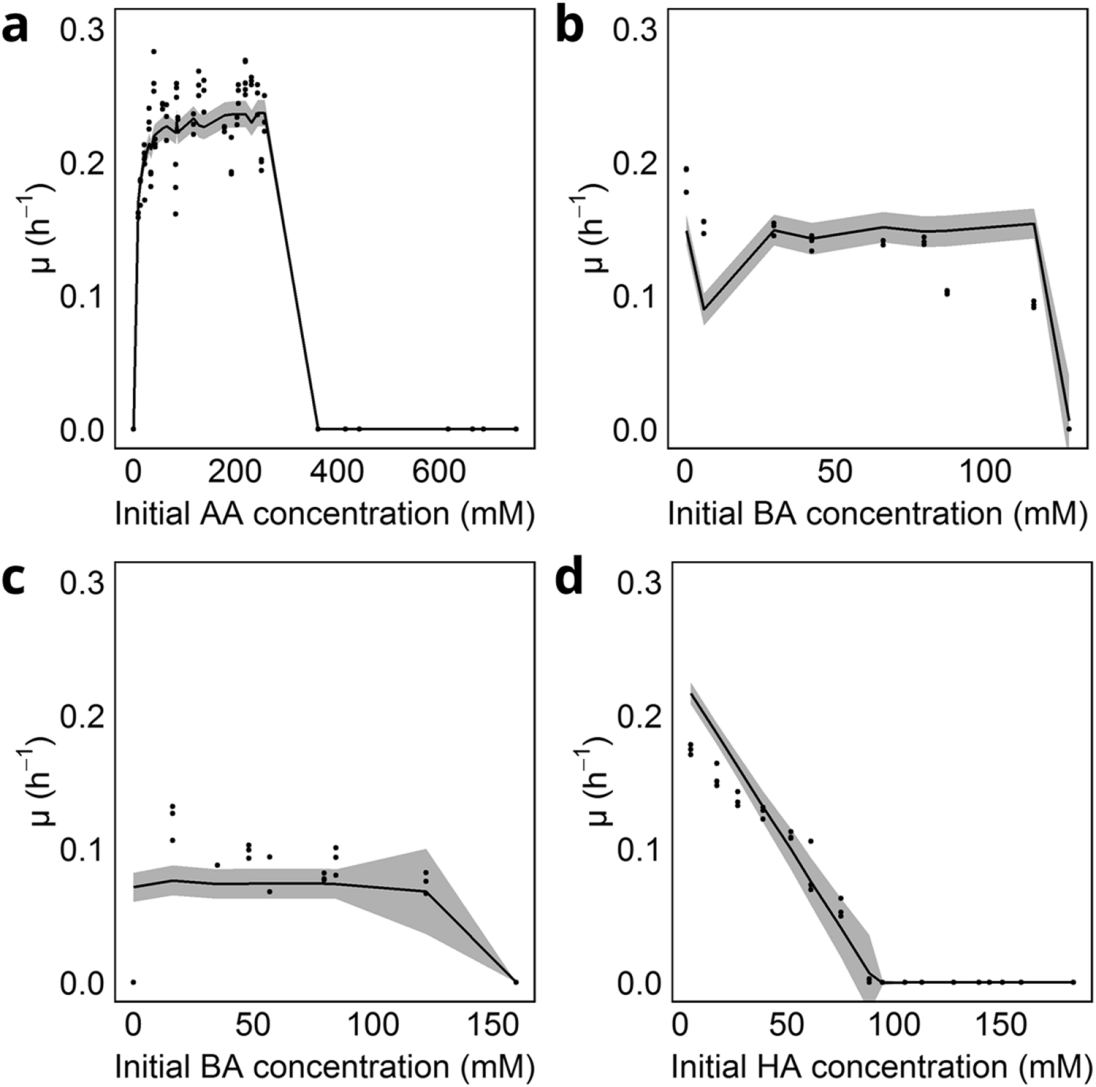
Fit and uncertainty on kinetic model for *C*. *kluyveri*. In all figures, dots represent a single experiment, with all experiments performed in triplicate. Full lines represent model output, shaded area is the uncertainty of the model. 3 A shows the fit to varying AA between 0 and 750 mM at a fixed concentration of EtOH (343 mM) (experiments A and B; Table 1). 3B shows µ in function of varying BA concentrations at a fixed initial EtOH concentration (343 mM) and AA-concentration (6 mM) (Experiment E; Table 1); 3 C similar to 3B but at an initial AA-concentration of 1.6 mM (Experiment F; Table 1). Lastly, 3D shows fit to Experiment G, varying concentrations of HA at standard DSM52 conditions (in practice 80 mM AA, 343 mM EtOH).

Parameter estimation: All parameters of the selected models (µ_max_, K_S,AA_, K_I,BA_ and K_HA_) were estimated by optimising the fit between the combined model and the entire dataset. This study obtained a µ_max_ that was slightly lower than what has been reported in literature (0.24 vs 0.28 h^−1^; Table 1). This might be due to differences in kinetic behaviour in batch vs. chemostat systems for *C*. *kluyveri*^7^, which has been suggested to be related to mixing^12^. The half-saturation index of 3.8 ± 0.9 mM for AA (K_S,AA_) is very close to the K_S,AA_ (3.5 mM) that was reported previously^8^ (Table 1). The same report estimates a K_S,BA_ of 3.5 mM BA in their review, obtained by calibrating a model with literature data, while in this experimental work no BA half-saturation constant could be determined due to the requirement of AA. Lastly, K_HA_ corresponds with an upper toxicity concentration of 91.3 ± 10.8 mM, in experiments with an initial pH between 7.4 and 8.2. When only protonated HA, instead of all HA, was considered the upper limit becomes 0.18 ± 0.01 mM protonated HA. Despite deviations in specific cases, the general trends observed in the experimental data are well matched by the output of the kinetic model (Fig. 4). Identifiability issues, i.e. multiple combinations of parameters resulting in the same model performance, appear to be limited, as indicated by moderate 95% confidence intervals (Table 1) and correlation factors between parameters (Supplementary Information S.2.8.).

**Table 1:** Parameter estimations for the combined kinetic model calibrated to data from experiments A, B, E, F and G simultaneously.

	This study	Literature	Reference
µ_max_ (h^−1^)	0.24 ± 0.01	Over 0.287 ± 0.008^#^	^7^
K_S,AA_ (mM)	3.8 ± 0.9	3.5	^8^
K_S,BA_ (mM)	—	3.5	^8^
K_I,BA_ (mM)	124.7 ± 5.7	—	—
K_HA_ (mM^−1^)	10.9 ± 1.3*10^−3^	—	—

Values reported for this study are the estimates ± 95% confidence intervals, estimated by linear approximation of the covariance matrix with the inverse of the Fisher Information Matrix.

^#^See S3.9 for details.

### Dynamic mass-balance model for validation of kinetic model

A dynamic mass-balance model was developed as a means for validating the kinetic model (Section 2.2.), using parameters from the kinetic model. This model was validated by comparing it to experimental data tracking substrate consumption and product formation in 96-WP (Supplementary Information S.2.10.). Lag time was removed from the data and simulations were run from the actual start of growth, as lag times were not considered in the dynamic model. The BA and EtOH half-saturation indices (K_S,BA_, K_S,EtOH_) were taken from literature^8^ because neither could be determined in this work.

**Figure 5:**
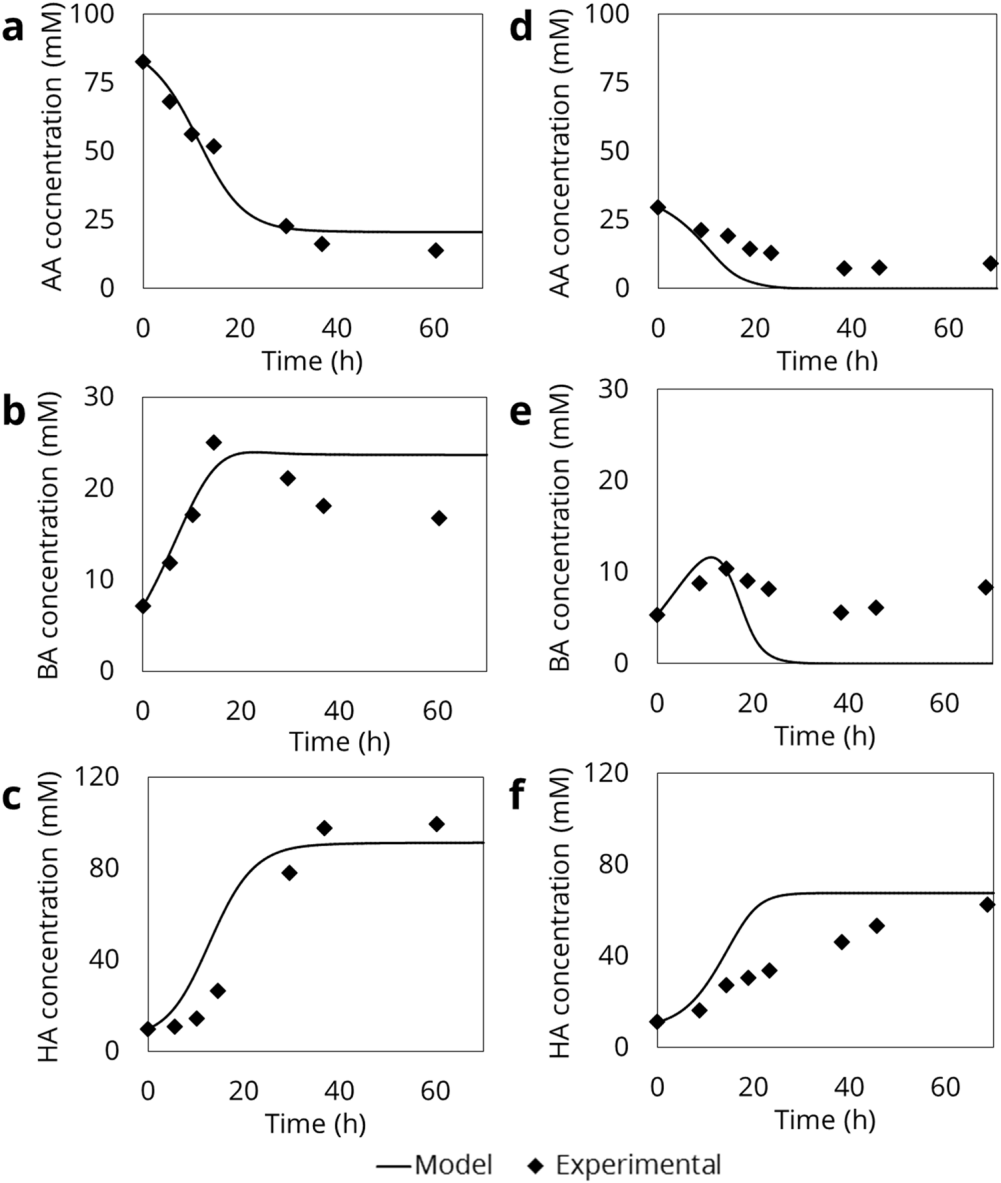
Model validation of dynamic ODE-model for HA-production by *C*. *kluyveri*. In all figures, diamonds represent experimental data, full lines represent model data. Figures a–c show results for growth at high AA-concentration (307 mM EtOH, 82.7 mM AA), with a, b and c showing AA, BA and HA respectively. Figures d–f show results for growth at low AA-concentration (219 mM EtOH, 29.6 mM AA), with d, e and f showing AA, BA and HA respectively.

The first simulated condition was based on the standard DSM52 medium (after lag time: 308 mM EtOH and 83 mM AA). Simulations of this experiment gave an overall good fit for each of the compounds (Fig. 5a–c). The second condition, at lower AA-concentrations (after lag time: 220 mM EtOH and 30 mM AA) diverged from experimental data (Fig. 5d–f), due to the dynamic ODE-model predicting complete consumption of substrates, while this was not observed in the experiments. This implies that, while the model can give reasonable predictions, at least some mechanisms are still missing in the model.

## Discussion

### Advantages and disadvantages of the high-throughput technique

The high-throughput technique described in this study can establish K_S_-values for the growth of anaerobic micro-organisms, however its main strength lies in the quantification of inhibitory behaviour. This is evidenced by the determination of inhibitory concentrations of the carboxylic acids involved in the metabolism of *C*. *kluyveri*. The determination of substrate affinity by OD may be hampered by the low sensitivity of the technique. More sensitive techniques, such as flow cytometry (accurate single cell counts) or by using a rotating disk electrode (single metabolite turnover), capable of establishing kinetic rates at lower biomass concentrations can be applied and integrated in the same data analysis framework^13,14^.

Generally, the performance of the cultures was similar in 96-WP compared to the classic method for cultivation, yet deviations occurred mainly at high substrate concentrations. These could be due to: (i) the different geometries of the incubation environments, i.e. Balch tubes are larger and deeper than wells in a 96-WP, potentially creating mass transfer limitations when biomass settles during static incubation, (ii) the lower H_2_-partial pressure in the covered, but unsealed 96-WP – H_2_ can equilibrate with the headspace of the anaerobic closet – compared to the sealed Balch tubes – H_2_ can only accumulate (hydrogen partial pressure is known to influence growth of *C*. *kluyveri*^15^, which has been hypothesised to be due to thermodynamical limitations^1^), (iii) the transfer of EtOH between wells in the 96-WP (Supplementary Information S.2.1.), e.g. from wells without growth to those with growth, further stimulating HA-production. Because of (iii), kinetic parameters could not be determined for EtOH, and all parameters are determined for an initial EtOH concentration of 343 mM. For future applications, it may be advisable to use a plastic film to seal the 96-WP, which has been shown to successfully prevent migration of EtOH in an abiotic experiment (Supplementary Information S.2.1.).

Regardless of the mechanism, the 96-WP methodology and data analysis framework – or a further optimised version of it – provides a foundation to assist in monitoring and optimising the MCCA-production process, and can in principle be applied to other microbial bio-production processes, for example succinic acid fermentation. It can, for instance, be used to reduce the effort required in selecting the best candidate from a range of pure cultures or enriched communities performing the same process. Alternatively, it can be used to scan operational conditions, e.g. pH, nutrients, optimum substrate concentrations, etc., with a minimum of effort, especially in comparison to conventional methods for kinetic parameter estimation. In order to attain the same data resolution in batch experiments would require intensive sampling campaigns (every 15 minutes over 5 days), while chemostats would require on-line monitoring and running many conditions over a long time, or in many parallel systems. Ultimately, this method provides a tool for fast parameter estimation, which can then be further tested in more applied conditions.

### Anionic MCCA can also exert toxicity

HA was shown to linearly inhibit the activity of *C*. *kluyveri*, with an upper toxicity limit of 91.3 ± 10.8 mM HA, or 0.18 ± 0.01 mM undissociated HA, strongly deviating from reported literature data: 7.5 mM undissociated HA for a mixed culture at pH 5.5^16^, 0.88 mM undissociated HA for a co-culture of *C*. *kluyveri* and *C*. *autoethanogenum*^17^, and 0.30 mM undissociated HA for an unadapted strain of *E*. *coli*^18^, all higher than what is reported here. The protonophoric behaviour of the undissociated acid – i.e. the funnelling of protons inside the cell and lowering intracellular pH – has been proposed as a mechanism for toxicity^19^. However, at a pH between 7.4 and 8.2, as was the case in these experiments, the potential for such behaviour appears low.

Next to the protonophoric behaviour, MCCA can also be toxic through modification of membrane properties by insertion into the membrane, enabled by its hydrophobic tail^20^. This behaviour could extend to the anionic form, considering the similar structure of anionic MCCA and the phospholipids composing the cell membrane – i.e. a hydrophilic head with hydrophobic tail. This insertion can cause increased membrane fluidity, leakage and possibly disruption of the electron transport chain for energy generation^20^. Royce *et al*. quantified this effect, observing a linear increase of membrane leakage and simultaneous linear decrease of µ in *E*. *coli* at increasing concentrations of octanoic acid^18^, very similar to the linear inhibition effect observed in *C*. *kluyveri* by HA in this study.

It should be noted that carboxylates show a stronger toxicity effect at lower pH^18^, limiting the interpretation of the determined toxicity limit for HA. The toxic concentration reported here is only valid for *C*. *kluyveri* DSM555 growing at pH 7 and above. Beyond that, these experimental results show that anionic MCCA-species are not harmless to micro-organisms. In this respect, the extrapolation of a toxicity limit for HA – expressed in concentration of undissociated acid – determined for a mixed culture at pH 5.5 to a pure culture at pH 7^8^, may be inaccurate. There is value in the reporting of toxicity limits of MCCA, but caution should be taken when interpreting these results, and pH and (un)dissociated concentrations of MCCA should be considered in this interpretation. Further work should quantify how strong the toxicity effects of both species of HA are, and whether they work independently or synergistically. Regardless of the exact mechanism of toxicity, the observation that both species of MCCA can be harmful to the biocatalyst implies HA should be extracted *in-situ* to prevent product toxicity.

### A cell is more than a bag of enzymes: why the assumption of *C*. *kluyveri* as a near-pure bio-catalyst does not hold

Several important experimental observations can be made that exposes the conflict between model and experimental data: (i) production of HA can continue even after net growth has stopped (Supplementary Information S.2.10), (ii) *C*. *kluyveri* does not consume all AA and BA present, even if there is still an electron donor (EtOH) present and the product (HA) is below toxic levels, and (iii) the experiment with a high AA concentration clearly shows a peak in BA-concentrations (Fig. 5b), indicating consumption of BA (Eq. (2)) can be faster than production of BA (Eq. (1)), before AA becomes limiting.

The first observation – production of HA with no observed net growth – can be explained by considering the assumptions made in the stoichiometric mass-balance model. In this model, the biomass acts as a near-pure catalyst; besides growth, no cellular processes – such as maintenance, decay, etc. – were taken into consideration. In the stationary phase, growth equals decay, implying the metabolism is still active and cells are still producing HA, a process not taken up in the current model structure, and explaining the observed discrepancy between model and reality.

The second observation of residual substrate is part of a larger story on the HA-metabolism and how *C*. *kluyveri* uses and transports substrates. It was observed in this study that *C*. *kluyveri* cannot ferment EtOH and BA without AA, confirming previous work^3^. In that study, 10 mM of AA was added to ‘kick-start’ the fermentation, while in the present study concentrations as low as 1.6 mM AA are shown to be sufficient to start growth. This trait cannot be incorporated in a purely enzymatic approach to modelling of HA-production. Considering the longer chain length of BA, compared to AA, one assumes that more energy is need to transport BA into the cell. This can imply that for a fresh culture of *C*. *kluyveri* to transport BA inwards and metabolise it, *C*. *kluyveri* first needs to consume AA to provide energy for this transport step. This hypothesis appears to be supported by µ remaining constant at increasing BA-concentrations, despite the presence of excess substrate. However, this does not explain leftover AA and BA at the end of the experiment. Another unknown mechanism might be at play governing both behaviours.

Lastly, assuming the energetics and enzymatics of BA and HA-production are the same^1,21^, there would be no reason to observe a net consumption of BA, unless AA is limiting. However, this and previous work^4^ have observed net BA-consumption with concentrations of AA well above K_S,AA_. This behavior might originate from the cell wall as a transport threshold. Studies have shown that product concentrations can be very different inside vs. outside bacterial cells^22^. Because of these varying concentrations, internal ratios of products and substrates might shift due to this transport threshold. A shift in the internal BA:AA-ratio, for instance, could affect the reaction rates – assuming a fixed affinity for both substrates by the enzymes – causing a temporary net consumption of BA, as cells move towards a new equilibrium BA:AA-ratio. On top of that, a pH gradient exists over the cell membrane to allow energy generation. This implies that the determined parameters are not valid for single enzyme kinetics, but aggregate the entire cell metabolism in one “apparent” kinetic parameter. Based on the available data, it would be highly complex to incorporate these processes in the model, as they are dependent on a delicate balance between many processes: active and/or passive transport of EtOH, AA and BA into the cell, active and/or passive transport of BA and HA out of the cell, conversion of AA and BA with generation of protons inside the cell, energy recovery using the pH gradient over the membrane, etc. As is the case for any biotechnological process, the understanding of fluxes – both of intracellular production and transport in and out of the cell – will be crucial for understanding and optimising this process^23^.

### An incomplete model’s potential

The model presented in this study attempted to experimentally determine kinetic parameters for *C*. *kluyveri* and use these to model the production of MCCA. This study started from the simplest model on a cellular level, i.e. growth as the only cellular process. However, simulations deviated from experimental results, demonstrating some processes need to be included in the model, such as the aforementioned membrane transport processes. The thermodynamic impact of H_2_ on growth kinetics has also not been incorporated, and might be relevant when moving towards simulations of large-scale processes where H_2_ does not immediately diffuse away. Previous studies have shown mixed communities can outperform the pure culture *C*. *kluyveri*^24,25^, showing the value of expanding the model to a complex, mixed community that produces MCCA. In that case, syntrophic reactions with H_2_-consumers (e.g. homoacetogens or hydrogenotrophic methanogens) and competition for substrates should also be incorporated. For these proposed expansions, the model presented in this study is a starting point, and strengthening the model will allow its use for the development of new applications.

## Conclusions

This study has demonstrated a novel, anaerobic high-throughput technique for analysis of the kinetic properties of micro-organisms, applied to *C*. *kluyveri* as an industrially relevant model organism. This technique allowed determination of growth rates (µ_max_, 0.24 ± 0.01 h^−1^), half-saturation indices (K_S,AA_, 3.8 ± 0.9 mM) and toxicity concentrations (AA, 330 mM; BA, 124.7 ± 5.7 mM; HA, 91.3 ± 10.8 mM HA). The developed framework for data-analysis was coupled to a biokinetic model of HA-production by *C*. *kluyveri*. This approach exposed knowledge gaps on how *C*. *kluyveri* handles substrates - specifically AA and BA -, how transport of substrates and products occurs on a cellular level and how MCCA exert their toxicity. These processes need to be thoroughly explored to come to a complete mechanistic model for process optimisation.

Future research should further explore this tool to approach a complete process model of the metabolism of *C*. *kluyveri*, in order to unlock the knowledge required to optimise a process towards the desired products, particularly when product inhibition is a factor. In parallel, this tool can also be used in kinetics-based process optimisation to benchmark novel bioproduction strains against existing organisms, monitor kinetic properties in bioreactors, study mixed culture fermentations, where pathway interactions will be crucial to give a meaningful interpretation to the obtained results.

Ultimately, the strength of this tool does not come from either the methodology to collect data, or the modelling, but from coupling the two, and provides a rapid and effective method to collect and interpret large amounts of data into kinetic parameters and a biokinetic model.

## Methods

### Culture and Media

*C*. *kluyveri* DSM555 was obtained from the German Collection of Microorganisms and Cell Cultures (DSMZ, Braunschweig, Germany) and was routinely cultivated in 40 mL DSM 52 medium in 120 mL serum flasks (Supplementary Information S.1.1.). Fresh medium was always inoculated with 10% (v/v basal medium %) grown culture. All experiments and routine cultivations were performed at 37 °C. Experiments used an inoculum first grown for approx. 60 h in DSM 52 at 37 °C. Axenity of the used culture was regularly evaluated at the start of each experiment by microscopy^2^.

### Experimental determination of growth parameters

#### Experiments for kinetic parameter estimation

To characterise the kinetics of *C*. *kluyveri*, a high-throughput growth curve technique was adapted from literature^11^, by which the growth of *C*. *kluyveri* was monitored under varying conditions. The obtained data was then used to determine growth rate (µ) under each of the experimental conditions, which in turn could be used to estimate kinetic parameters.

First, Balch tubes^26^ containing varying concentrations of one substrate or product were prepared by adding this compound from a sterile, anaerobic stock after autoclaving of the medium. The Balch tubes were inoculated with an active *C*. *kluyveri*-culture. A sample from these Balch tubes was taken to analyse initial conditions and pH, (constant throughout all experiments at 7.85 ± 0.14). Subsequently, these Balch tubes were used to prepare a 96-well plate (Greiner Bio-One Cellstar^®^, sterile, flat-bottom, transparent) (96-WP) in an anaerobic (10% CO_2_, 90% N_2_) closet (GP-Campus, Jacomex, TCPS NV, Rotselaar, Belgium). Each well of the 96-WP was filled with 200 µL of inoculated broth from a Balch tube, while ensuring sterile conditions. The outer wells (rows A and H, columns 1 and 12) of the 96-WP were filled with 300 µL of sterile, substrate-free medium, acting as an evaporation buffer. The 96-WP was covered by a sterile transparent lid to avoid contamination. Each experiment was performed in triplicate in the 96-WP, except where noted otherwise. Growth was monitored by measuring optical density at 620 nm (OD) at 15 min intervals using a spectrophotometer (Tecan Sunrise, Grödig, Austria) inside the anaerobic closet, which was temperature controlled at 37 °C. As a positive control, the Balch tubes were statically incubated at 37 °C.

The aim and conditions of each experiment are outlined in Table 2. For experiments B, C and G (Table 2), Balch tubes were inoculated directly from the routinely cultivated *C*. *kluyveri* culture. For experiments with low substrate concentrations (experiments A, D, E and F; Table 2), 40 mL of routinely cultivated *C*. *kluyveri* culture was transferred to a sterile 50 mL Falcon^®^ tube, closed under anaerobic conditions in an anaerobic closet and subsequently centrifuged for 8 min at 8610 g outside the anaerobic closet. Supernatant was removed in the anaerobic closet and cells were resuspended in the same volume of substrate-free DSM52-medium. This resuspended culture was used to inoculate the Balch tubes to be used for preparation of the 96-WP. Low redox values of the medium were not fully maintained during centrifugation as indicated by coloration of resazurin^27^. This was not an issue as indicated by unchanged lag times and final maximum product concentrations compared to non-centrifuged cultures inocula.

#### Yield determination

Ethanol growth yield (Y_EtOH_) of *C*. *kluyveri* DSM555 was determined by analysing the increase in biomass concentration as Volatile Suspended Solids (VSS) of a culture grown until the late exponential phase (48 h) in batch bottles (liquid volume = 0.2 L, n = 3) in standard DSM52 medium. Batch bottles were used to ensure sufficient volume for analysis of both initial and final biomass concentrations without affecting the experiment.

### Tracking of production kinetics for model validation

Monitoring OD in 96-WP gives information on biomass production throughout the growth period, but does not provide insights on substrate consumption or product formation. Therefore, as well as to validate the results of the modelling effort, product formation by *C*. *kluyveri* DSM555 in 96-WP was investigated in Experiments H and I (Table 2). Two conditions – 80 mM initial AA (standard DSM52-medium; Exp H) and 34 mM initial AA (low AA-concentration; Exp I) - were tracked by dividing the 96-well plate in 2 sections; the top 3 rows (rows B-D; High AA) vs. bottom 3 rows (rows E-G; Low AA). All wells within each section were filled with 200 µL of inoculated medium from the same source. Over 10 time points, 3 wells were sampled (following a pre-randomised scheme, Supplementary Information S.1.2.), and their content pooled. Emptied wells were refilled with 200 µL sterile DSM 52 medium to avoid increased evaporation from the remaining wells. This entire experiment was performed in triplicate, i.e. 3 96-WP, one inside the spectrophotometer measuring at 620 nm every 15 min, and the other two also in the anaerobic closet, under the assumption that incubation in the spectrophotometer has no influence on the growth of *C*. *kluyveri*.

### Analytical methods

For experiments A–G, pH in each well was measured at the end of the experiment (Consort SP28X, Turnhout, Belgium), and liquids of replicate conditions in a single 96-WP were pooled. For experiments H and I, pH of the pooled samples was analysed at each time point (Consort SP28X, Turnhout, Belgium). Pooled samples for all experiments were diluted 1:1 with demineralised water, filtered (0.20 µm) and stored (-20 °C) for later analysis of organic acids and alcohols. The remaining volumes, after incubation, of the corresponding Balch tubes were also stored similarly. Samples were appropriately diluted before analysis.

Carboxylic acids and alcohols were determined by gas chromatography with flame ionization detector (Supplementary Information S.1.5.)

VSS analyses were performed according to Standard Methods 2540D and E^28^.

### Model development

#### Calculation of growth rates (µ)

To calculate growth parameters from the growth curve data, corrections were applied in accordance with literature:^29^ OD was corrected with the average of the uninoculated samples (${\rm{\Delta }}OD$) and subsequently calculating $\mathrm{ln}(\frac{\Delta {OD}}{\Delta {O}{{D}}_{{\rm{\min }}}})$ with ${\rm{\Delta }}O{D}_{{\rm{\min }}}$ being the initial OD in each well. This log-transformation is important to estimate µ correctly, as the most commonly used population growth equations were developed using this parameter^29^. Additionally, the log-transformation increases the importance of early exponential phase growth, before substrate limitation or product inhibition can occur. Subsequently, the Richards equation (Eq. (3)) was fitted to this transformed data, using the *nls*.*lm* optimisation algorithm from the minpack.lm package in R^30,31^. This fit resulted in a µ, lag time (λ), carrying capacity (A) and $\nu$ - a shape factor with no biological meaning – for each well.

3 $$\mathrm{ln}(\frac{\Delta {OD}}{\Delta O{D}_{{\rm{\min }}}})=A\cdot {(1+\nu \cdot {e}^{1+\nu }\cdot {e}^{\frac{\mu }{A}\cdot {(1+\nu )}^{1+\frac{1}{\nu }}\cdot (\lambda -t)})}^{-\frac{1}{\nu }}$$

#### Kinetic model

Several equations for modelling growth kinetics, based on substrate and/or product concentrations, were selected from literature^32–34^ or developed in this work based on experimental observations (Table 3). They were chosen because (i) their limited number of parameters would ensure maximum identifiability during calibration, and (ii) model parameters have a mechanistic meaning. The parameters used in these models are: (i) µ_max_, the maximum growth rate; (ii) K_S,_ the half-saturation index, i.e. the lower concentration where µ is half of µ_max_; (iii) K_I_, the half-saturation inhibition index, i.e. the upper concentration where µ is half of µ_max_; (iv) K, the linear inhibition constant, i.e. the relative rate at which µ decreases with increasing concentrations of the inhibitory compound.

**Table 2:** Overview of anaerobic high-throughput experiments for determination of kinetic properties of *C*. *kluyveri*.

Exp ID	Experiment description^#^	EtOH	AA	BA	HA	Donor:Acceptor^~^
		mM	mM	mM	mM	—
A	Low AA	343^*^	**0**–**257**	<1	<0.1	1.3–∞
B	High AA	343	**0**–**750**	<4	<4	0.5–∞
C	EtOH inhibition	**168 – 1764**	60	<2	<10	0.1–30.6
D	BA	343	0	**0**–**113**	0	3.0–∞
E	BA with 1.6 mM AA	343	1.6	**0**.**4**–**128**	<1	2.8–212.4
F	BA with 6 mM AA	343	6	**0**–**160**	<1	2.8–51
G	HA inhibition	343	80	<3	**5**–**184**	3.8–4.6
H	Product formation – high AA	343	80	1.8	7.6	4.2
I	Product formation – low AA	343	34	1.7	7.4	9.6

Bold text indicates substrate or product concentration varied in experiments A–G (cf. experiment description).

^#^Experiment description indicates which substrate or product concentration was targeted or what the goal of the experiment was.

^*^All concentrations are actual initial concentrations as determined from the balch tubes at the start of the experiment.

^~^Donor:acceptor is the ratio of electron donor (EtOH) to electron acceptor (AA + BA) at the start of the experiment.

**Table 3:** Overview of all kinetic models used for model comparison.

Name	Equation	Eq.	Ref.
Substrates			
Monod	$\mu ={\mu }_{{\rm{\max }}}\cdot \frac{S}{{K}_{{\rm{S}}}+S}$	(4)	^33^
Haldane	$\mu ={\mu }_{{\rm{\max }}}\cdot \frac{S}{{K}_{{\rm{S}}}+S+\frac{{S}^{2}}{{K}_{{\rm{I}}}}}$	(5)	^32^
Monod + toxicity limit	$\mu ={\mu }_{{\rm{\max }}}\cdot \frac{S}{{K}_{{\rm{S}}}+S}\cdot (1-\frac{1}{1+{{\rm{e}}}^{-({\rm{S}}-{{\rm{K}}}_{{\rm{I}}})}})$	(6)	This study
Products			
Toxicity limit	$\mu ={\mu }_{{\rm{\max }}}\cdot (1-\frac{1}{1+{{\rm{e}}}^{-({\rm{P}}-{{\rm{K}}}_{{\rm{I}}})}})$	(7)	This study
Linear inhibition	$\mu ={\mu }_{{\rm{\max }}}\cdot (1-K\cdot P)$	(8)	^34^

S indicates substrate concentration, P refers to product concentrations, µ_max_ is the maximum growth rate, K_S_ is the half-saturation index, K_I_ is half-saturation inhibition concentration and K is the linear inhibition effect.

For model selection, these equations were calibrated to the experimental growth rate data for substrate (AA – Experiments A and B) and products (BA – Experiments E and F, HA – Experiment G), after which the best model was selected per compound, by comparing residuals of the fits for each model. For BA, the effect of the supplemented AA was taken into consideration by adding a Monod-term, with the K_S,AA_ obtained in the model selection of AA. In a second step, the selected models were combined through multiplication, and all parameters were calibrated simultaneously on all data sets (Experiments A, B, E, F and G) using the previously obtained estimates as initial values for the optimisation algorithm. Parameters were estimated in Python, using the pyIDEAS package^35^, using the Nelder-Mead algorithm for parameter optimisation. To estimate the 95% confidence intervals, the inverse of the Fisher Information Matrix was calculated to obtain a linear approximation of the covariance matrix^36^.

#### Dynamic mass-balance model

To validate the kinetic model described in section 4.5.2., a dynamic mass-balance model was developed. This model simulates substrate consumption, product formation and biomass growth over time based on a set reaction stoichiometry. In this study, a fixed two-step stoichiometry was used – cf. reactions (1) and (2) –, assumed to take place unidirectionally at reaction rates r_1_ and r_2_, respectively. This results in a set of ordinary differential equations (ODE), which can, generally, be written as follows:

9 $$\frac{{\rm{d}}S}{{\rm{d}}t}=-{a}_{s}\cdot {r}_{1}-{b}_{s}\cdot {r}_{2}$$

In this equation, a_S_ is the stoichiometric coefficient from reaction (1) and b_S_ the stoichiometric coefficient from reaction (2) for compound S. Comparing this to the conventional way of modelling substrate consumption by micro-organisms in a one-step reaction (X being the biomass concentration, µ the growth rate and Y the yield for this reaction):

10 $$\frac{{\rm{d}}S}{{\rm{d}}t}=\frac{\mu \cdot X}{Y}$$

The reaction rates can be rewritten as ${r}_{i}=\frac{{\mu }_{i}}{{Y}_{i}}\cdot X$, index *i* indicating reaction 1 or 2. Assuming Y_1_ and Y_2_ – the yield per reaction – is the same for both reactions (since ATP-generation is the same for both reactions^21^), and converting these to Y_EtOH_ (${Y}_{EtOH}=\frac{1}{6}\ast {Y}_{1}=\frac{1}{6}\ast {Y}_{2}$) - the mass balance eq. (11) can be rewritten as:

11 $$\frac{dS}{dt}=(\frac{{a}_{S}\cdot {\mu }_{1}}{6\cdot {Y}_{EtOH}}+\frac{{b}_{S}\cdot {\mu }_{2}}{6\cdot {Y}_{EtOH}})\cdot X$$

Considering the short term of the experiments conducted here (max. 90 hours), biomass decay was assumed to be negligible, yielding the following ODE for evolution of biomass:

12 $$\frac{dX}{dt}=({\mu }_{1}+{\mu }_{2})\ast X$$

For modelling of µ_1_ and µ_2_, the kinetic model was applied here, which structurally, yields the following equations, where each term is the most appropriate model according to the model selection as described in section 4.5.2.:

13 $${\mu }_{1}={\mu }_{{\rm{\max }}}\cdot ({Affinity}\,AA)\cdot ({Toxicity}\,AA)\cdot ({Affinity}\,EtOH)\cdot ({Toxicity}\,EtOH)\cdot ({Toxicity}\,BA)\cdot ({Toxicity}\,HA)$$

14 $${\mu }_{2}={\mu }_{{\rm{\max }}}\cdot ({Affinity}\,BA)\cdot ({Toxicity}\,BA)\cdot ({Affinity}\,EtOH)\cdot ({Toxicity}\,EtOH)\cdot ({Toxicity}\,AA)\cdot ({Toxicity}\,HA)$$

Additionally, due to one micro-organism catalysing both reactions, an additional weight factor is introduced, to balance the observed growth rate as the weighted average of both calculated growth rates:

15 $${w}_{i}=\frac{{\mu }_{i}}{{\mu }_{1}+{\mu }_{2}}$$

Ultimately, the generalised mass balance equations become:

16 $$\frac{dS}{dt}=(\frac{{a}_{S}\cdot {w}_{1}\cdot {\mu }_{1}}{6\cdot {Y}_{EtOH}}+\frac{{b}_{S}\cdot {w}_{2}\cdot {\mu }_{2}}{6\cdot {Y}_{EtOH}})\cdot X$$

17 $$\frac{dX}{dt}=({w}_{1}\cdot {\mu }_{1}+{w}_{2}\cdot {\mu }_{2})\ast X$$

Using Eqs 13–17 (with Eq. 16 applied for each modelled substrate or product), a system of ODEs was implemented in Python using the pyIDEAS package^35^.

## Electronic supplementary material

Supplementary information to: A novel high-throughput method for kinetic characterisation of anaerobic bioproduction strains, applied to Clostridium kluyveri

## References

[1] Angenent LT (2016). Chain Elongation with Reactor Microbiomes: Open-Culture Biotechnology To Produce Biochemicals. Environ. Sci. Technol..

[2] Barker HA, Taha M (1941). Clostridium Kluyverii, an organism concerned in the formation of caproic acid from ethyl alcohol. J. Bacteriol..

[3] Bornstein BT, Barker HA (1948). The Energy Metabolism Of Clostridium kluyveri And The Synthesis Of Fatty Acids. J. Biol. Chem..

[4] Thauer RK, Jungermann K, Henninger H, Wenning J, Decker K (1968). The Energy Metabolism of Clostridium kluyveri. Eur. J. Biochem..

[5] Seedorf H (2008). The genome of Clostridium kluyveri, a strict anaerobe. Proc. Natl. Acad. Sci. USA.

[6] Wang S, Huang H, Moll J, Thauer RK (2010). NADP+ reduction with reduced ferredoxin and NADP+ reduction with NADH are coupled via an electron-bifurcating enzyme complex in Clostridium kluyveri. J. Bacteriol..

[7] Kenealy WR, Waselefsky DM (1985). Studies on the substrate range of Clostridium kluyveri; the use of propanol and succinate. Arch. Microbiol..

[8] Cavalcante WDA, Leitão RC, Gehring TA, Angenent LT, Santaella ST (2017). Anaerobic fermentation for n-caproic acid production: A review. Process Biochem..

[9] Donoso-Bravo A (2011). Model selection, identification and validation in anaerobic digestion: A review. Water Res..

[10] Wett B, Schoen M, Phothilangka P, Wackerle F, Insam H (2007). Model-based design of an agricultural biogas plant: application of Anaerobic Digestion Model No. 1 for an improved four chamber scheme. Water Sci. Technol..

[11] Geirnaert A (2014). Butyricicoccus pullicaecorum, a butyrate producer with probiotic potential, is intrinsically tolerant to stomach and small intestine conditions. Anaerobe.

[12] Hegner R, Koch C, Riechert V, Harnisch F (2017). Microbiome-based carboxylic acids production: from serum bottles to bioreactors. RSC Adv..

[13] Van Nevel S, Koetzsch S, Weilenmann HU, Boon N, Hammes F (2013). Routine bacterial analysis with automated flow cytometry. J. Microbiol. Methods.

[14] Prévoteau, A. *et al*. Hydrodynamic chronoamperometry for probing kinetics of anaerobic microbial metabolism - case study of Faecalibacterium prausnitzii. *Sci*. *Rep*. **5**, (2015). 10.1038/srep11484 PMC4486957 26127013

[15] Schoberth S, Gottschalk G (1969). Considerations on the Energy Metabolism of Clostridium kluyveri. Arch. Microbiol..

[16] Ge S, Usack JG, Spirito CM, Angenent LT (2015). Long-Term n-Caproic Acid Production from Yeast-Fermentation Beer in an Anaerobic Bioreactor with Continuous Product Extraction. Environ. Sci. Technol..

[17] Diender M, Stams AJM, Sousa DZ (2016). Production of medium - chain fatty acids and higher alcohols by a synthetic co-culture grown on carbon monoxide or syngas. Biotechnol. Biofuels.

[18] Royce LA, Liu P, Stebbins MJ, Hanson BC, Jarboe LR (2013). The damaging effects of short chain fatty acids on Escherichia coli membranes. Appl. Microbiol. Biotechnol..

[19] Palmqvist E, Hahn-Hägerdal B (2000). Fermentation of lignocellulosic hydrolysates. II: Inhibitors and mechanisms of inhibition. Bioresource Technology.

[20] Desbois AP, Smith VJ (2010). Antibacterial free fatty acids: activities, mechanisms of action and biotechnological potential. Appl. Microbiol. Biotechnol..

[21] Spirito CM, Richter H, Rabaey K, Stams AJ, Angenent LT (2014). Chain elongation in anaerobic reactor microbiomes to recover resources from waste. Curr. Opin. Biotechnol..

[22] Péquignot C, Dussap C-G, Pons A, Gros J-B (1997). Intra- and extracellular concentrations of glutamate, lactate and acetate during growth of Corynebacterium glutamicum on different media. J. Ind. Microbiol. Biotechnol..

[23] Blank LM (2017). Let’s talk about flux or the importance of (intracellular) reaction rates. Microb. Biotechnol..

[24] Gildemyn S (2017). Upgrading syngas fermentation effluent using Clostridium kluyveri in a continuous fermentation. Biotechnol. Biofuels.

[25] Grootscholten TIM, Steinbusch KJJ, Hamelers HVM, Buisman CJN (2013). Improving medium chain fatty acid productivity using chain elongation by reducing the hydraulic retention time in an upflow anaerobic filter. Bioresour. Technol..

[26] Balch WE, Wolfe RS (1976). New Approach to the Cultivation of Methanogenic Bacteria: 2-Mercaptoethanesulfonic Acid (HS-CoM)-Dependent Growth of Methanobacterium ruminantium in a Pressurized Atmosphere. Appl. Environ. Microbiol..

[27] Tratnyek PG (2001). Visualizing Redox Chemistry: Probing Environmental Oxidation-Reduction Reactions with Indicator Dyes. Chem. Educ..

[28] APHA. *Standard methods for the examination of water and wastewater*. *American Public Health Association* (2005).

[29] Begot C, Desnier I, Daudin JD, Labadie JC, Lebert A (1996). Recommendations for calculating growth parameters by opticaldensity measurements. J. Microbiol. Methods.

[30] R Core Team. R: A language and environment for statistical computing. (2015).

[31] Timur, E. V., Mullen, K. M., Spiess, A.-N. & Bolker, B. minpack.lm: R Interface to the Levenberg-Marquardt Nonlinear Least-Squares Algorithm Found in MINPACK, Plus Support for Bounds. at https://cran.r-project.org/package=minpack.lm (2015).

[32] Sivakumar A, Srinivasaraghavan T, Swaminathan T, Baradarajan A (1994). Extended monod kinetics for substrate inhibited systems. Bioprocess Eng..

[33] Monod J (1949). The Growth Of Bacterial Cultures. Annu. Rev. Microbiol..

[34] Hinshelwood, C. N. *The Chemical Kinetics Of The Bacterial Cell*. (Clarendon Press, 1952).

[35] Van Daele T, Van Hoey S, Nopens I (2015). pyIDEAS: an Open Source Python Package for Model Analysis. in. Comput Aided Chem Eng.

[36] Omlin M, Reichert P (1999). A comparison of techniques for the estimation of model prediction uncertainty. Ecol. Modell..

