# Supplementary material for: A novel high-throughput method for kinetic characterisation of anaerobic bioproduction strains, applied to Clostridium kluyveri
Source: Sci Rep. 2018 Jun 27;8:9724. doi: 10.1038/s41598-018-27594-9 (PMC6021416; doi:10.1038/s41598-018-27594-9)
Supplement: Supplementary file 1 — Supplementary information to: A novel high-throughput method for kinetic characterisation of anaerobic bioproduction strains, applied to Clostridium kluyveri [file 41598_2018_27594_MOESM1_ESM.pdf]

Supplementary information to: A novel high-throughput method for kinetic characterisation of anaerobic bioproduction strains, applied to *Clostridium kluyveri*

Pieter Candry[1], Timothy Van Daele[2], Kyrina Denis[1,2], Youri Amerlinck[2], Stephen J. Andersen[1], Ramon Ganigué[1], Jan B.A. Arends[1], Ingmar Nopens[2], Korneel Rabaey[1]

[1] Center for Microbial Ecology and Technology (CMET),

[2] BIOMATH, Department of Mathematical Modelling, Statistics and Bioinformatics,

Faculty of Bioscience Engineering Ghent University, Coupure Links 653, 9000 Gent, Belgium

* Correspondence to: Korneel Rabaey, Ghent University; Faculty of Bioscience Engineering; Centre for Microbial Ecology and Technology (CMET); Coupure Links 653; B-9000 Gent, Belgium; phone: +32 (0)9 264 59 76; fax: +32 (0)9 264 62 48; E-mail: Korneel.Rabaey@UGent.be; Webpage: www.cmet.UGent.be.

S.1. Materials & Methods

S.1.1. Culture & Media

Routine cultivation medium was prepared in accordance to DSM52, with following recipe (in 1L basal medium): 0.31 g K_2HPO_4, 0.23 g KH_2PO_4, 0.25 g NH_4Cl, 0.20 g $MgSO_4.7H_2O$, 1.00 g yeast extract, 10.00 g K-acetate and 20 mL $EtOH.L^{-1}$. Medium was boiled and subsequently cooled under N_2-atmosphere, followed by distribution over 10 mL balch tubes or 40 mL serum flasks. The headspace of the balch tubes or serum flasks was then replaced with N_2 using a gas headspace exchange apparatus. After autoclaving vitamins and trace elements were added (0.33 mL per 10 mL of medium), made by adding 1 mL of trace element solution SL-10, 1 mL of selenite-tungstate solution and 0.1 mL of 7-vitamin solution to 30 mL of 84 g/L $NaHCO_3$ and filter-sterilizing the solution under N_2-atmosphere. The SL-10 trace element solution consisted of (in 1L stock solution): 10 mL 7.7M HCl, 1.5 g $FeCl_2.4H_2O$, 0.07 g $ZnCl_2$, 0.15 g $MnCl_2.4H_2O$, 0.006 g H_3BO_3, 0.19 g $CoCl_2.6H_2O$, 0.002 g $CuCl_2.2H_2O$, 0.024 g $NiCl_2.6H_2O$ and 0.036 g $Na_2MoO_4.4H_2O$. The selenite-tungstate solution contained (in 1L stock solution): 0.5 g NaOH, 0.003g $Na_2SeO_3.5H_2O$ and 0.004 g $Na_2WO_4.2H_2O$. The 7-vitamin solution consisted of (in 100 mL stock solution): 0.1 g vitamin B_{12}, 0.08 g p-aminobenzoic acid, 0.02 g D(+)-Biotin, 0.2 g nicotinic acid, 0.1 g Ca-pantothenate, 0.3 g pyridoxine hydrochloride, 0.2 g thiamine-$HCl.2H_2O$. Before inoculation a solution with reducing agents was containing 5 $g.L^{-1}$ of L-cysteine-$HCl.H_2O$ and 5 $g.L^{-1}$ of $Na_2S.9H_2O$, dissolved in basal medium, using anaerobic aseptic techniques.

S.1.2. Production kinetics

A randomized sampling scheme (Figure S.1) was constructed using R[1]. One scheme (Figure S.1) was made for 3 rows and 10 columns, and applied to both conditions in the 96-WP. This experiment was performed in triplicate, i.e. 3 96-WP, of which one was in the platereader for continuous readings (620 nm) every 15 min. Actual time into the experiment for each timepoint is given in Table S.I.

	1	2	3	4	5	6	7	8	9	10	11	12
A	Evaporation Buffer											
B		2	4	1	5	10	3	8	6	6	7	
C		4	1	10	8	9	3	8	7	5	9	
D		2	5	7	9	3	2	6	1	10	4	
E		2	4	1	5	10	3	8	6	6	7	
F		4	1	10	8	9	3	8	7	5	9	
G		2	5	7	9	3	2	6	1	10	4	
H												

Figure S.1. Randomized sampling scheme for monitoring of production kinetics in 96-WP. Colours indicate different initial conditions; in the gray area, initial substrate concentrations were 343 mM EtOH and 81 mM AA, for blue area this was 343 mM EtOH and 34 mM AA. The number in each well indicates at which timepoint that well was sampled.

Table S.I. Time into the experiment per timepoint at which wells were sampled according to scheme in Figure S.1

Timepoint	Time (h)
0	0.0
1	9.2
2	13.4
3	22.4
4	31.1
5	36.7
6	41.2
7	45.6
8	60.7
9	68.0
10	91.3

S.1.3. Abiotic EtOH-evaporation experiments

Because of the volatility of EtOH, an abiotic experiment was set up to establish whether EtOH can transfer from wells with high concentrations to those with lower concentrations within a single 96-WP. One 96-WP was divided into several sections (Figure S.2), with rows containing DSM52 medium with or without EtOH to test whether transfer was taking place through the headspace. Three sections can be distinguished within this 96-WP. A first section (columns 10-11) allowed to investigate whether EtOH could transport from one well to another. Columns 2-6 could show transfer of EtOH further than one row, implying transport through the headspace. Lastly, column 8 acted as a negative control, i.e. it was expected no EtOH could migrate through the physical separation by petroleum jelly.

To test whether a plastic seal film (Nunc® Multiwell Plate polyolefin sealing tape, Sigma-Aldrich) could relieve EtOH evaporation issues through the headspace, an additional experiment was performed (Figure S.3). In this experiment, also the wells in the rim of the 96-WP were used to test whether this could enhance the high-throughput nature of the method even further. The rationale behind the experiment was much the same: columns 2-4 and 10-11 were added to see whether EtOH could migrate between adjacent wells, while columns 6 and 8 were added to test EtOH migration over longer distances. Wells within one condition were pooled at the end of the experiment and EtOH concentration was determined of the pooled sample. No experiment with petroleum jelly border was added, because the seal was used as a physical barrier between wells in the experiment.

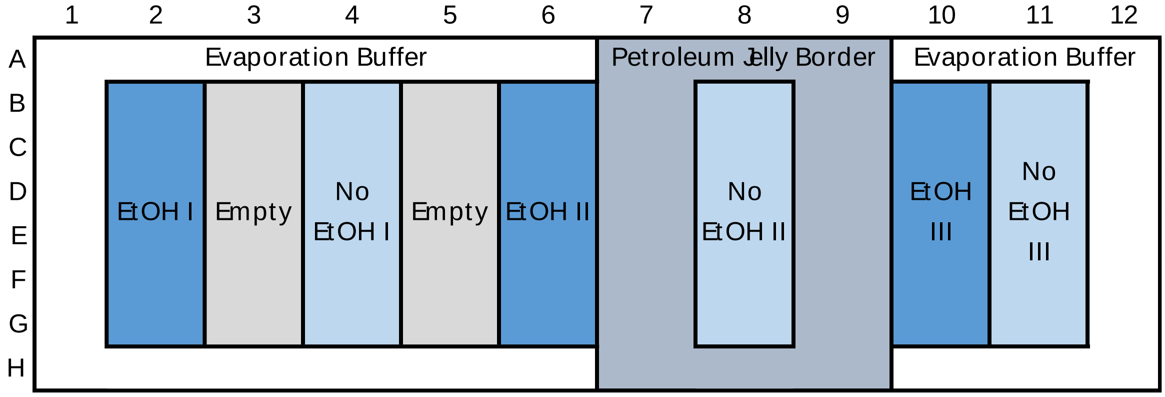

Figure S.2. Schematic representation of 96-well plate for abiotic EtOH-evaporation experiment.

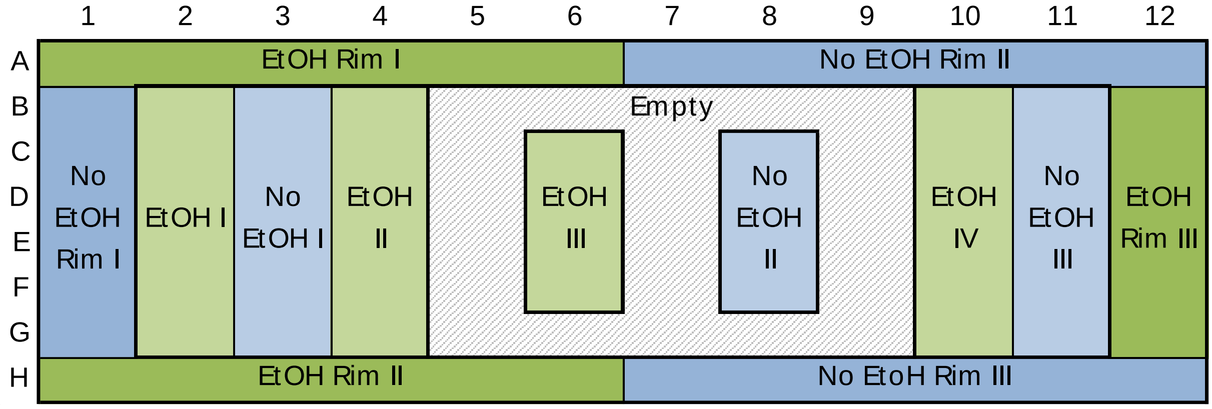

Figure S.3. Schematic representation of 96-well plate for abiotic EtOH-evaporation experiment using plastic film seal

S.1.4. Linking OD to biomass concentrations

Quantifying biomass based on OD can give valuable information, considering the large amount of OD data available in the high-throughput growth curve experiments. To link these parameters, a culture grown on standard DSM52 medium in 200 mL batches was taken after 2 and 4 days. These cultures were centrifuged for 8 min at 8610g, supernatans was removed and filtered over 0.20 µm filters to obtain cell-free spent medium. This spent medium was first used to resuspend the centrifuged biomass in 1/5th of the original volume and subsequently make a six-fold serial dilution (1:1 cell suspension:spent medium). For each dilution, total cell count, VSS and OD were determined. Total cell counts were determined by flow cytometry[2]. This experiment was performed in triplicate, i.e. a dilution series was made from 3 independently

grown cultures on day 2 and day 4.

S.1.5. Analytical methods

C2 to C8 carboxylic acids (including isoforms C4 to C6) were determined by gas chromatography (GC; GC-2014, Shimadzu®, The Netherlands), with a DB-FFAP 123-3232 column (30 m × 0.32 mm × 0.25 µm; Agilent, Belgium) and a flame ionisation detector (FID). Liquid samples were conditioned with 2 mL sulfuric acid, 200 mg sodium chloride, and 2-methyl hexanoic acid as an internal standard for quantification before further extraction with diethyl ether (1:1 volume sample:ether). The sample (1 µL) was injected at 250°C with a split ratio of 50 and a purge flow of 3 mL min^{-1}. The oven temperature increased by 10°C min^{-1} from 110 to 250°C where it was maintained for 5 min. The FID had a temperature of 300°C. Nitrogen carrier gas was maintained at a flow rate of 2.49 mL min^{-1}. The GC was externally calibrated with a minimum detection limit of 60 mg/L for AA, and 10 mg/L for all higher carboxylic acids.

Alcohols (acetone, methanol, ethanol, 1-propanol, 2-propanol, 1-butanol, 1-pentanol and 1-hexanol) were measured by GC (GC-2010-Plus, Shimadzu®, Belgium) with a Stabilwax-DE-S column (30m x 0.32mm x 1.0 µm, Shimadzu®, Belgium) and an FID. 1 µL of sample was injected at 230°C with a split ratio of 50 and purge flow of 3.0 mL min^{-1}. Oven temperature was put at 35°C for one minute, after which it increased from 35°C to 230°C at a rate of 10°C min^{-1} where it was kept for 2 minutes. FID temperature was set at 250°C, carrier gas was nitrogen gas at a flow rate of 1.09 mL min^{-1}. The GC was externally calibrated with a minimum detection limit of 25 mg/L.

S.1.6. Model development for chemostat simulations

Ordinary differential equations (ODE) described in main text (Section 3.2.) were modified for simulation of chemostat conditions. The modified equations for substrate consumption or product formation (eq. (S1)) and biomass production (eq. (S2)) are given below.

$$\frac{dS}{dt} = \left(\frac{a_S * w_1 * \mu_1}{6 * Y_{EtOH}} + \frac{b_S * w_2 * \mu_2}{6 * Y_{EtOH}}\right) * X + D * (S_{in} - S) \quad \text{(S1)}$$

$$\frac{dX}{dt} = (w_1 * \mu_1 + w_2 * \mu_2 - D) * X \quad \text{(S2)}$$

For simulation of chemostat experiments[3], the reported conditions were applied to the model (see Table S.II). The concentrations of substrates (EtOH, AA) and products (BA, HA) reached after simulation for 500h of chemostat operation were then compared to the reported average concentration for each condition and plotted in Figure 4 of the main text.

Table S.II. Conditions applied to modified dynamic mass-balance model for simulation of chemostat conditions[3]

Condition nr.	EtOH influent (mM)	AA influent (mM)	D (h^{-1})
1	42.2±1.9	24.6±0.9	0.050±0.001
2	45.4±1.2	25.9±0.1	0.100±0.005
3	43.3±1.6	25.5±1.0	0.199±0.004
4	44.7±1.1	26.2±0.8	0.287±0.008
5	91.3±4.2	10.4±0.7	0.038±0.012
6	106.5±5.2	9.7±1.1	0.144±0.002
7	100.2±4.7	9.5±0.5	0.201±0.003
8	95.0±1.8	10.9±1.8	0.282±0.005

S.1.7. Thermodynamic calculations for assessment of influence of H_2 accumulation

H_2 accumulating in the headspace can limit the metabolism of *C. kluyveri* by reducing the Gibbs free energy (ΔG^1), i.e. the amount of energy liberated during the reaction. To evaluate the importance of this limitation, thermodynamical calculations were performed, using data and methods from literature[4]. Two situations were assessed: (1) ΔG^1 if only the first reaction step is considered, and (2) ΔG^0 if only HA is an end product, i.e. the sum of both reaction steps (see Table S.II.). For both situations, initial conditions were used cf. the DSM 52 medium, and ΔG^1 was calculated as the reaction progressed according to the stoichiometry of the reaction. The limiting substrate was used to assess the reaction progression, where the first reaction was limited by AA, and the second by EtOH availability.

To assess the impact of H_2, this calculation was performed with and without accumulation of H_2 in the headspace. To calculate accumulation of H_2, a Balch tube with a total volume of 26 mL was considered, with 11.83 mL of liquid medium. Futhermore it was assumed that: (i) no substrate was used for biomass production, (ii) no H_2 was lost, through diffusion or in the metabolism, (iii) all H_2 produced immediately dissolved from the liquid into the headspace, (iv) pH does not change during the reaction, and, (v) initially a very low H_2 partial pressure of 0.0001 atm (10.1 Pa) was present in the headspace. For the case without H_2 accumulation, H_2 partial pressure was assumed to stay constant at this same 0.0001 atm.

Table S.III. Reactions considered in thermodynamical calculations. ΔG^{01}_{T} is Gibbs free energy change at biological standard state (pH 7 at 37°C), calculated using ΔG^{0}_{f} and ΔH^{0}_{f} data and methods from literature[4].

Reaction stoichiometry	ATP produced[5]	ΔG^{01}_{T} (kJ/mol)	Max ΔG^{1} (kJ/mol)
6 EtOH + 4 acetate$^-$ → 5 butyrate$^-$ + H^+ + 2 H_2 + 4 H_2O	2.5#	-183.82	-180~
12 EtOH + 3 acetate$^-$ → 5 hexanoate$^-$ + 2 H^+ + 4 H_2 + 8 H_2O	5	-404.861	-360

# Number of ATP produced according to stoichiometric model of Angenent et al.[5]

~ Max ΔG^{1} calculated, assuming 72 kJ/mol ATP[5]

S.2. Results

S.2.1. Abiotic EtOH evaporation experiments

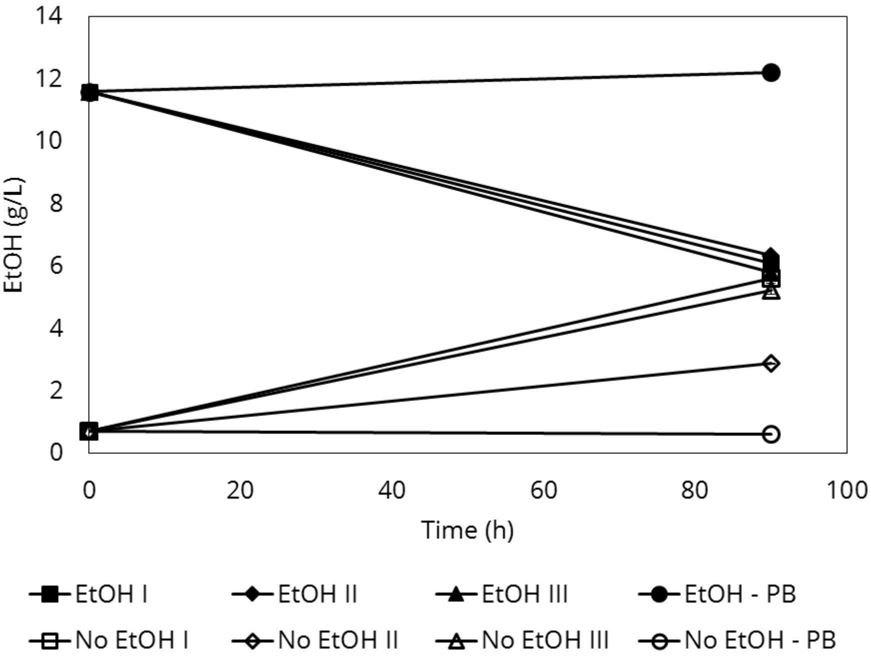

Figure S.4. Evolution of EtOH-concentration in abiotic 96-WP experiment.

An abiotic experiment following EtOH-concentrations within a 96-well plate confirms that EtOH can be transferred through the headspace, according to concentration gradients. It appears an equilibrium was established across all wells, and even in part through a physical barrier (petroleum jelly). Diffusion to the environment might occur too, but 91.9% of all EtOH initially present was recovered at the end of the experiment, not taking into account potential transfer to the wells acting as evaporation buffer. Evaporation appears not to be troublesome for organic acids, since (1) they usually behaved similarly in 96-WP and balch tubes, (2) no apparent stoichiometric inconsistencies were observed as was the case with EtOH, and (3) initial pH was 7.85±0.14, implying all organic acids to be present in their anionic, non-volatile form.

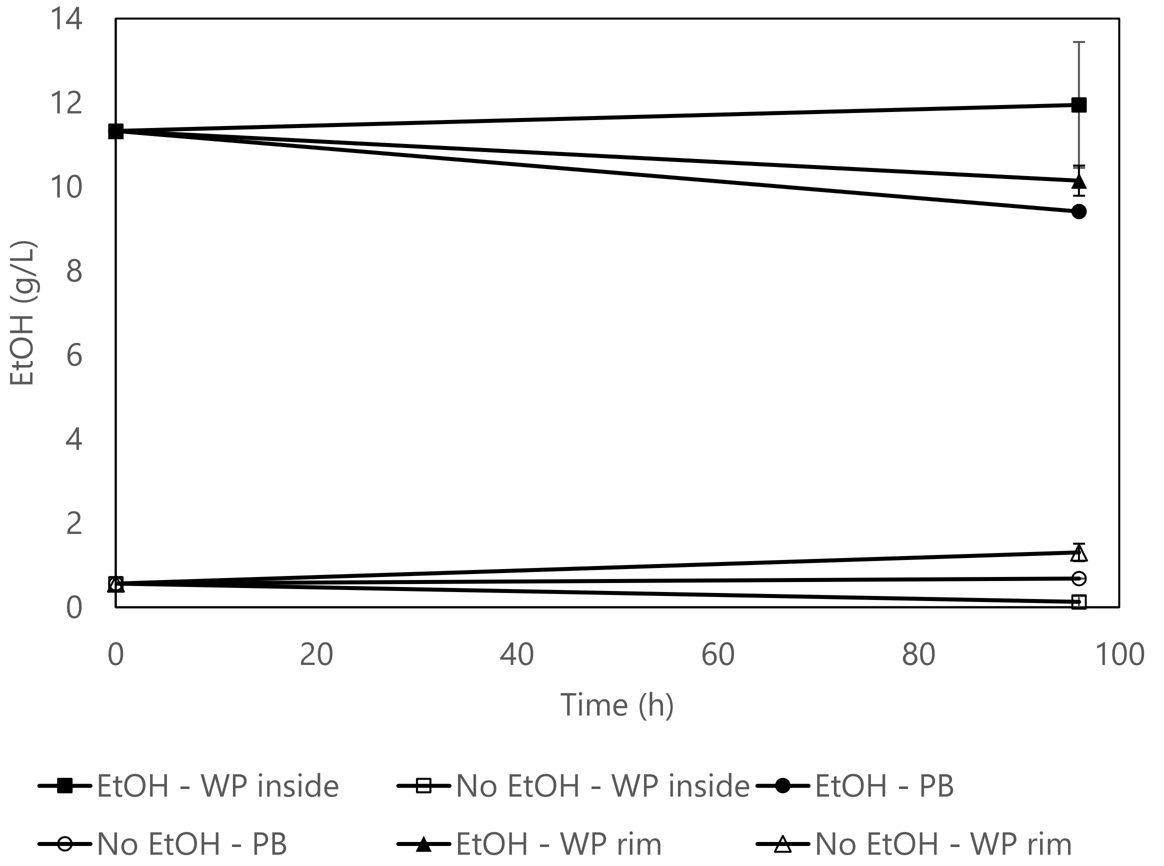

Figure 5. Evolution of EtOH-concentrations in abiotic 96-WP experiment using plastic film seals to prevent EtOH evaporation. Data represented is the average EtOH-concentration of the replicates (n=3), error bars indicate standard deviation.

When a plastic film seal was used to physically separate each well, EtOH no longer migrated between wells in an abiotic experiment. Furthermore, the seal also allowed the outer rims to be used without evaporation issues. The seal used in this experiment was not sterilized, yet no contamination of the medium was observed, likely due to the very restrictive medium compostion. When richer media are used, or axenity is strictly necessary, sterile films could also be used, but were not tested here.

S.2.2. Lag time (λ) as a function of EtOH-concentration

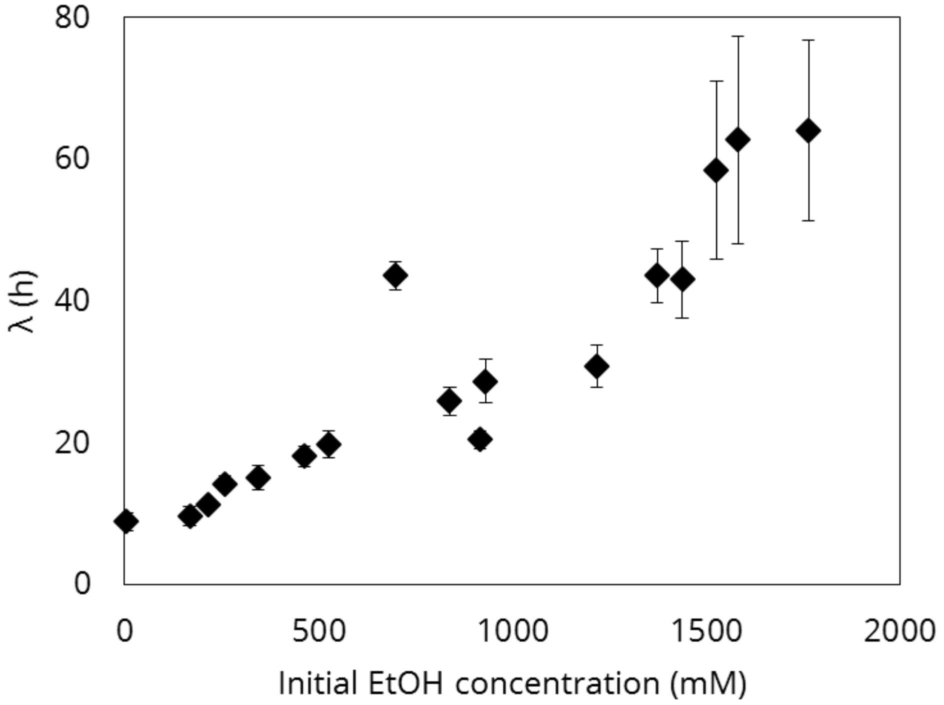

Figure S.6. Lag time (λ) as a function of the initial EtOH-concentration. Error bars represent standard deviation over replicates (n=3).

In Experiment C – varying the initial EtOH-concentration across the wells - a strong correlation (Pearson coefficient of 0.923) can be observed between λ and the initial EtOH-concentration in the broth (Figure S.6). A potential source of this correlation is the evaporation of EtOH from wells with high concentration and diffusion through the headspace to those wells with low EtOH-concentrations. When concentration drops low enough, growth starts, resulting in increasing λ with increasing concentrations. This hypothesis is corroborated by the results in S.3.1. showing EtOH can be transferred between wells.

S.2.3. EtOH:AA-ratio

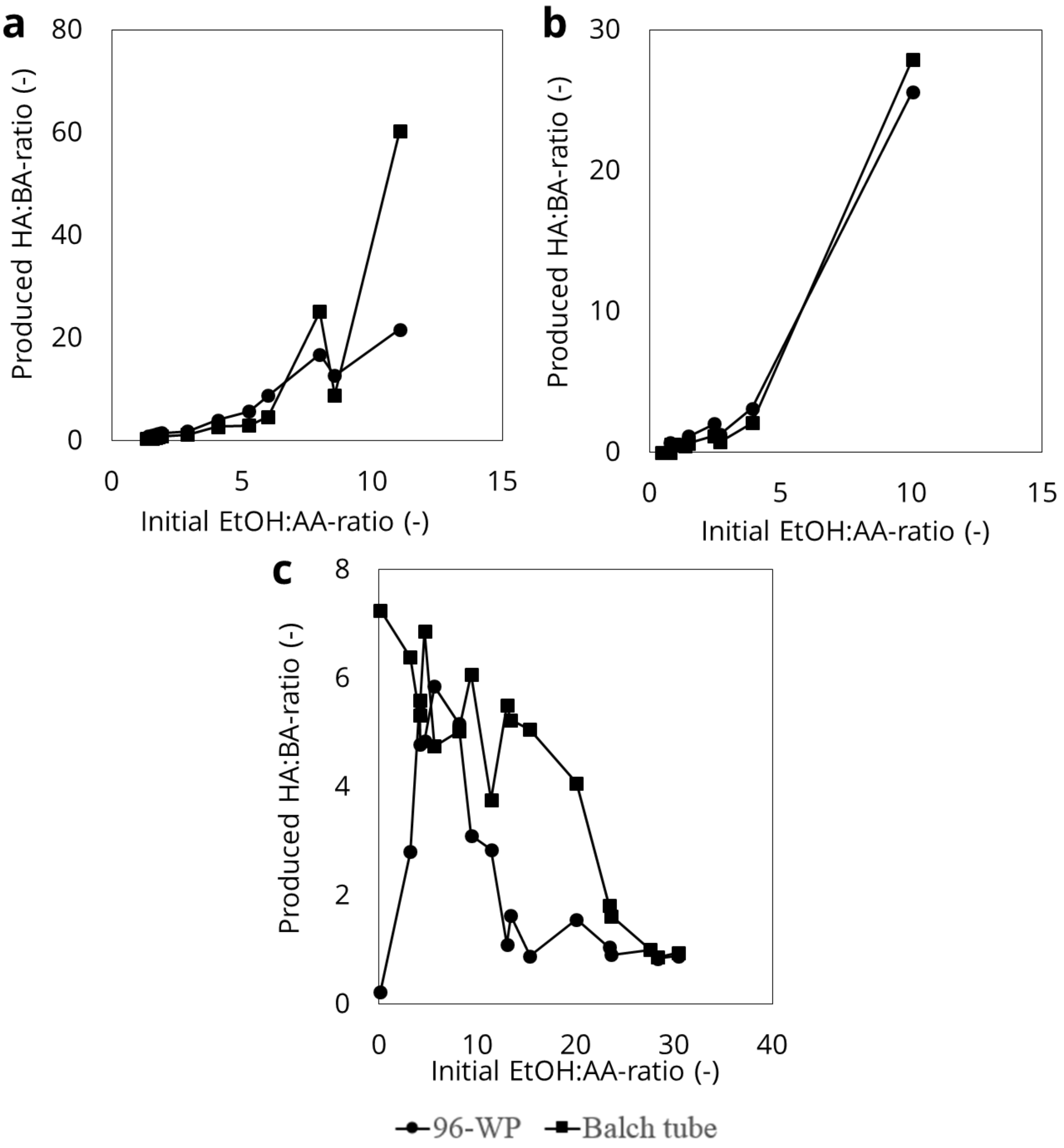

Figure S.7. Influence of initial ethanol (EtOH) to acetic acid (AA) donor on specificity of product output, expressed as the ratio of hexanoic acid (HA) produced to butyric acid (BA) produced. Full circles (●) represent results in Balch tubes, full squares (■) represent results in 96-WP. Only datapoints with net production of BA and HA in both 96-WP and Balch tubes are shown.

An increase in EtOH:AA-ratio increases the selectivity for HA-production, confirming earlier literature reports[6,7]. For Experiments A and B (Figure 1A and 1B, respectively), an increase in the EtOH:AA ratio – with fixed EtOH-concentration- results in an increase of the

HA:BA-ratio produced (Figure S.5A & S.5B). Similarly, fixing the AA-concentration, an increasing initial EtOH:AA-ratio again increases the HA:BA-ratio in the balch tubes at the end of Experiment C (Figure 1C). This is true up to the point where EtOH-toxicity becomes an issue, as indicated by the decreased production of BA and HA in balch tubes above an initial concentration of at most 700 mM EtOH (in this case an EtOH:AA-ratio of 10, Figure S.5C). This is confirmed by correlation coefficients for the HA:BA-ratio produced to the initial EtOH:AA ratio (Table S.IV).

Table S.IV. Pearson correlation coefficients between initial EtOH:AA-ratio and HA:BA-ratio produced over the experiment

	Experiment A	Experiment B
Balch tubes	0.82	0.96
96-WP	0.98	0.94

S.2.4. Correlating OD and biomass concentrations

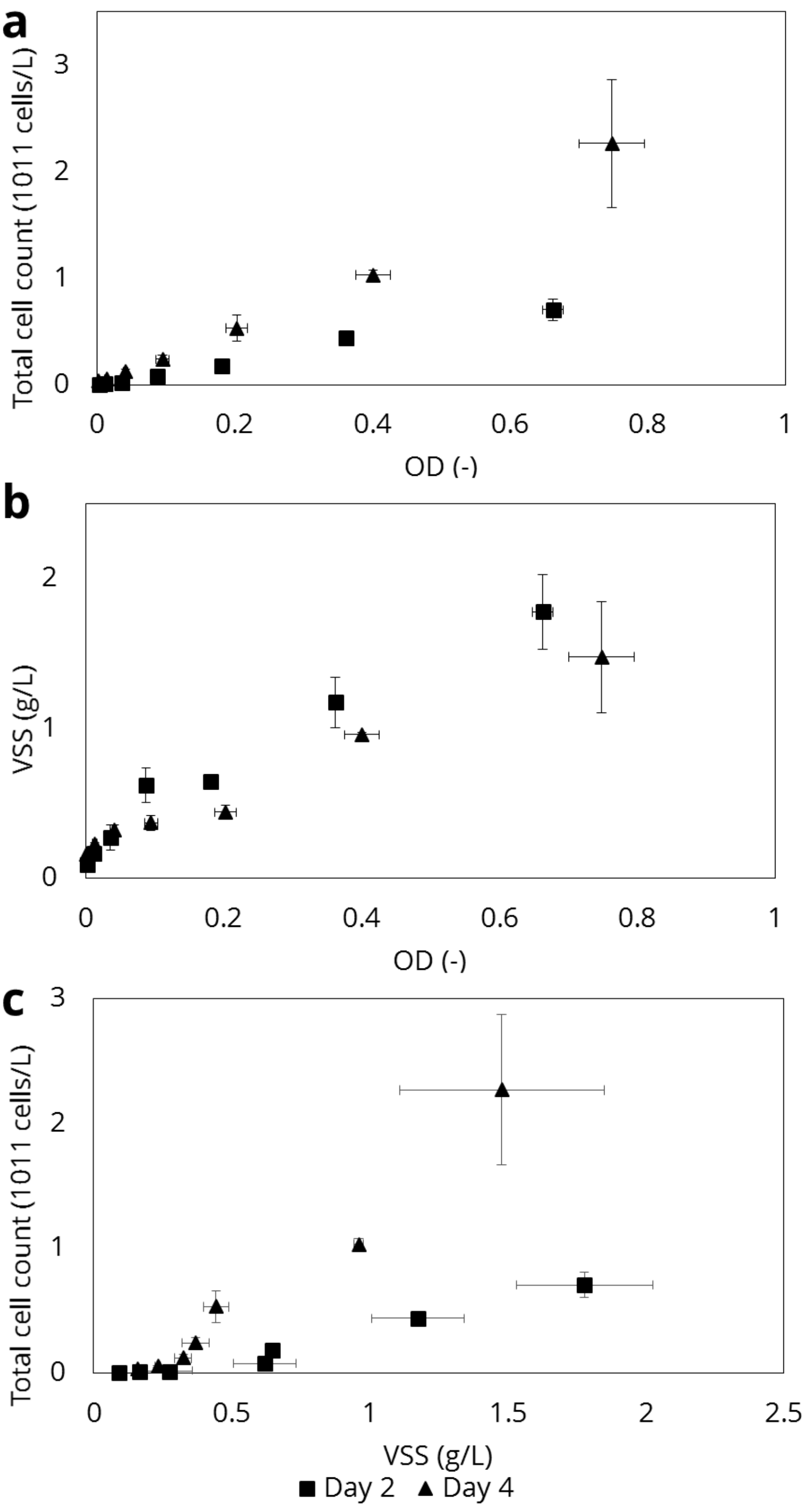

Figure S.8. Total cell count (A) and VSS (B) as a function of OD and total cell count as a function of VSS (C) after 2 (full squares, ■) and 4 (full triangles, ▲) days of growth in batch mode. Error bars are standard deviations over triplicates, error bars can be obscured by symbols.

In a dilution series experiment, an OD was linked to VSS and total cell counts – by flow cytometry – as well as VSS and total cell counts to each other. For a given OD, biomass concentrations expressed as VSS will be lower after 4 days than at 2 days. However, the opposite is true when looking at total cell counts; the same OD at 4 days implies more cells present than it does at 2 days. This apparent contradiction implies the amount of cells per g VSS increases between day 2 and 4 – confirmed by Figure S.8C – which can be explained by the sporulation behaviour of *Clostridium kluyveri* after 3 days[8]. Because of these shifts in the relationships between OD, VSS and total cell counts over time, quantifying biomass based on OD is not possible for *Clostridium kluyveri* for extended periods – i.e. when sporulation can occur.

S.2.5. Inconsistent growth curves in Experiment D

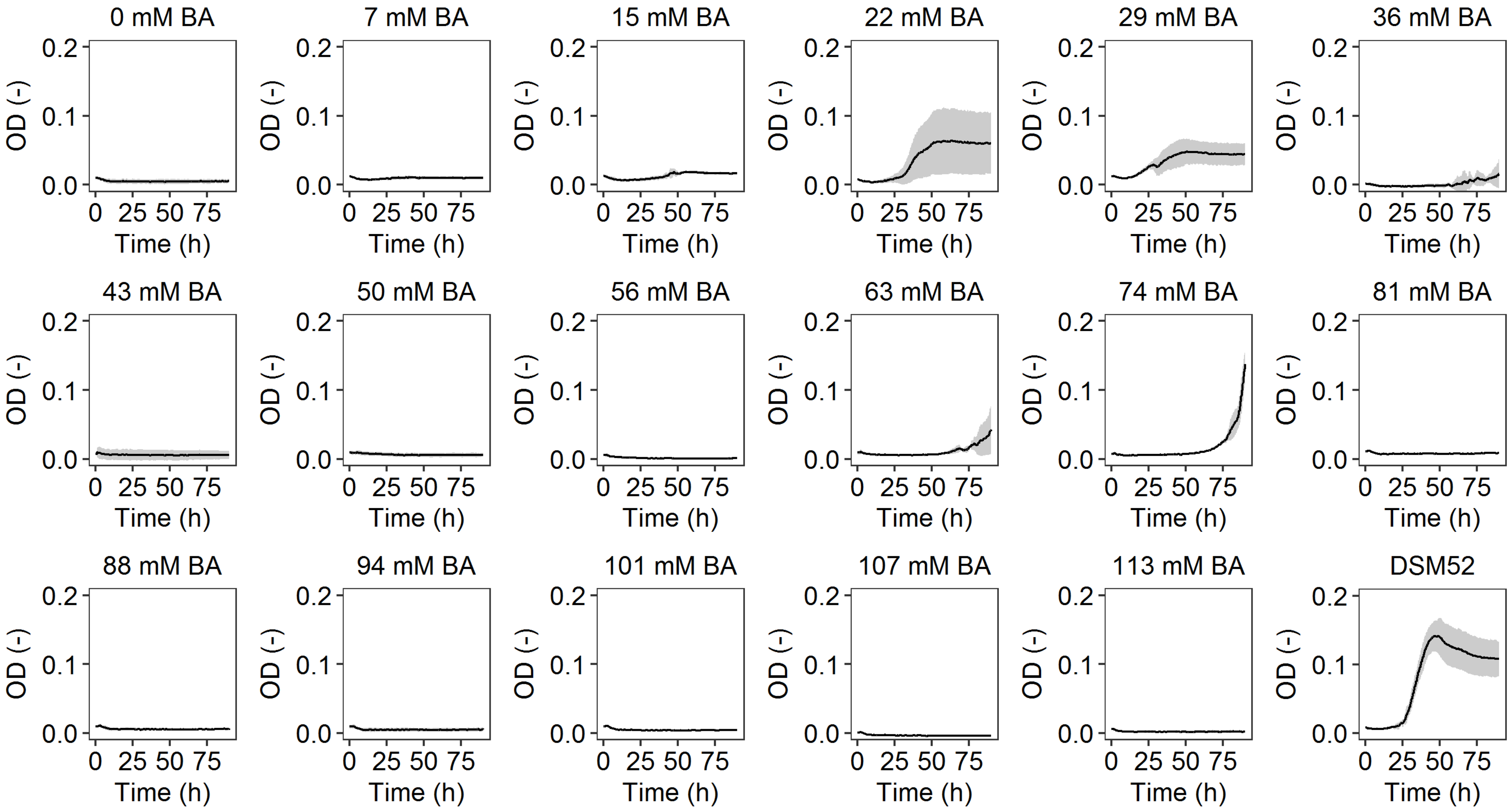

Figure S.9. Growth curves for all wells in Experiment D (varying BA, without supplemented AA), titles represent initially supplied concentration of BA, lines and grey ribbons respectively represent average and standard deviations over triplicates.

S.2.6. Model selection

The proposed models for each organic acid were calibrated using the data from the experiments for that acid, i.e. the proposed models for AA were calibrated using data from experiments A and B, BA using data from experiments E and F and HA using data from experiment G (Table 1, main text),. The calibrations of these models are summarised in Table S.V. The best model for each compound was selected based on the lowest residuals value of all models as well as a visual control of the fit to the data.

Table S.V. Overview of results for model selection for each organic acid involved in the metabolism. The parameter values reported in this table are only valid within the datasets for each organic acid and do not represent the overall best fit.

Organic acid	Model Type	μ_{max} (h^{-1})	K_S (mM)	K_I (mM)	K (mM^{-1})	Residuals value
AA	Monod	0.17±0.01	-2.07±1.4	-	-	566.6
	Haldane	No fit achieved				
	**Monod coupled to toxicity limit term***	**0.25±0.01**	**4.69±2.8**	**330 mM (arbitrarily chosen)**	**-**	**27.26**
BA	Linear inhibition	0.35±0.05			$5.4 \pm 1.1 \times 10^{-3}$	42.2
	**Toxicity limit**	**0.26±0.03**		**124.9±5.7**		**39.8**
HA	**Linear inhibition**	**0.19±0.03**			**$10.1 \pm 1.5 \times 10^{-3}$**	**2.8**
	Toxicity limit	0.14±0.02		61.6±1.4 mM		11.0

***Bold text indicates best fit**

Residuals are the sum of the difference between model prediction and experimental data for all data points

S.2.7. HA-toxicity

To confirm increased salinity was not the mechanism of HA-toxicity, a control experiment was included in experiment G (main text, Table I) – varying initial concentrations of HA and monitoring growth. NaCl was supplemented to the standard DSM52-medium to obtain the same electrical conductivity as the experiment with the highest HA-concentration. The average of the replicates, and standard deviations over replicates, of this NaCl-control are shown next to some selected growth curves at different HA-concentrations (including lowest and highest concentration) in Figure S.8. Even though the lag time is longer for the NaCl-control (24.7±2.4h) than for 5 mM HA (11.7±0.3h), and the maximum OD is lower (0.228±0.038 for NaCl vs. 0.407±0.005 for 5mM HA), there is still growth at an electrical conductivity equivalent to 184 mM HA, while growth is fully inhibited at HA concentrations above 95 mM.

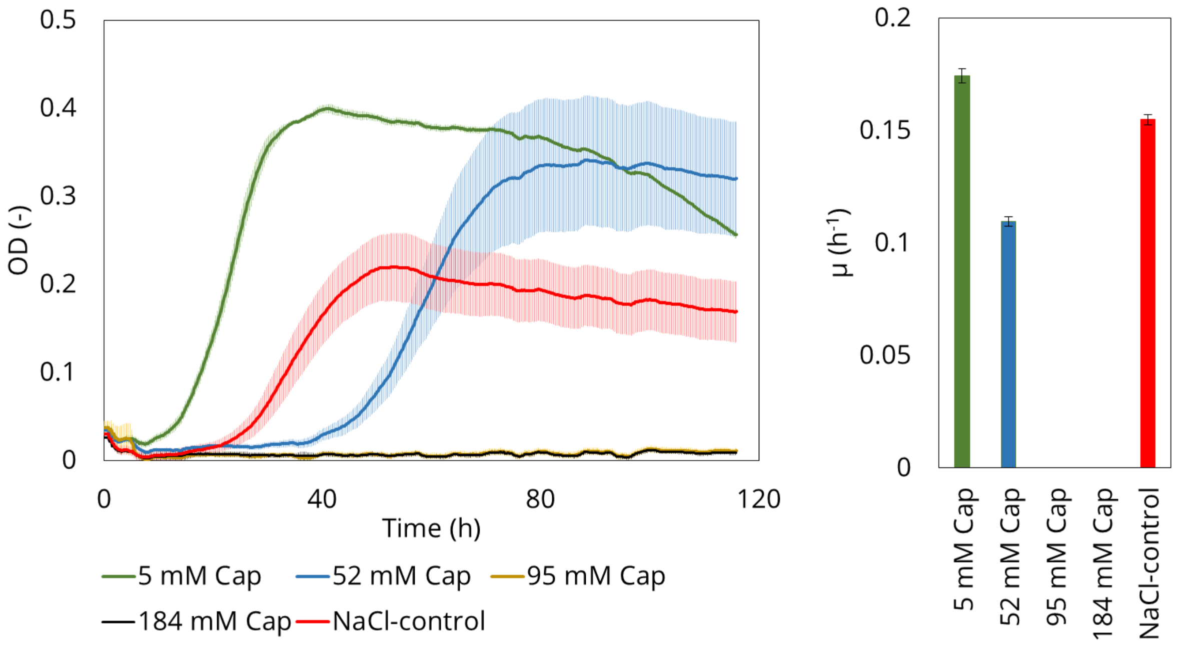

Figure S.10. Comparison of growth at different concentrations of HA with the NaCl-control put at the same electrical conductivity as the highest HA-concentration. All data obtained in experiment G. Error bars indicate standard deviation over all replicates for that condition (n=3).

S.2.8. Parameter estimation

Fit appropriateness of the kinetic model was evaluated by a combination of 95% confidence intervals (Table 3) – estimated by linear approximation of the covariance matrix with the inverse of the Fisher Information Matrix -, correlations between parameters (Table S.VI) and normality of the model output deviation (Figure S.11).

The correlation matrix is shown in Table S.VI. A moderate positive correlation is found between the affinity constant for AA ($K_{S,AA}$) and $\mu_{max,}$ whereas all other correlations are low. Hence, this should not lead to problems during parameter estimation.

Table S.VI. Correlation matrix for parameter estimation of kinetic model. Values given are Pearson coefficient for correlation between all parameters

	μ_{max}	$K_{S,AA}$	$K_{I,BA}$	K_{HA}
μ_{max}	1	0.579	0.057	0.162
$K_{S,AA}$	0.579	1	0.156	0.056
$K_{I,BA}$	0.057	0.156	1	0.002
K_{HA}	0.162	0.056	0.002	1

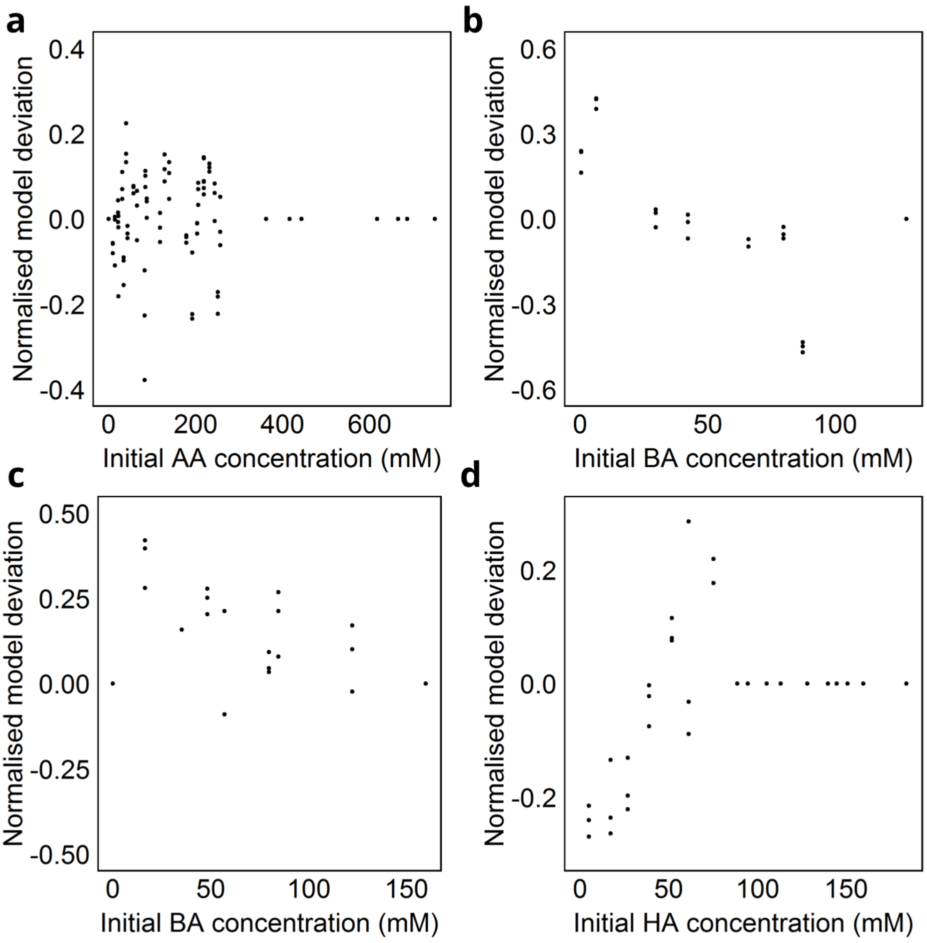

Figure S.11. Normalised deviation of model output for all organic acids involved in the metabolism of *C. kluyveri*. S.9A. shows the data for varying AA-concentration at a fixed EtOH concentration (343 mM) (experiments A and B; Table 1). S.9B shows model output deviation as a function of varying concentrations of HA with an initial AA-concentration of 6 mM (Experiment E; Table 1), while S.9C shows the same, but with 1.6 mM AA initially present. Lastly, S.9D shows normalized model output deviation with varying HA-concentrations in standard DSM52 medium (in practice: 80 mM AA, 343 mM EtOH).

S.2.9. Dynamic mass-balance model

A second model validation was performed by expanding the model to a chemostat reactor (see section S.3.1.). Simulations of this chemostat model were run under the same conditions as reported in literature[3] (Figure S.12). This confrontation hints at another shortcoming of the model: despite an apparent μ_{max} of 0.240±0.011 h^{-1} observed in batch growth experiments in this study, chemostat studies demonstrated that a culture of *Clostridium kluyveri* is not yet washed out at a D of 0.287±0.008 h^{-1} [3]. Continuous simulations of this dynamic stoichiometric mass balance model showed culture wash-out at a D as low as 0.187 h^{-1} at standard conditions (343 mM EtOH, 100 mM AA).

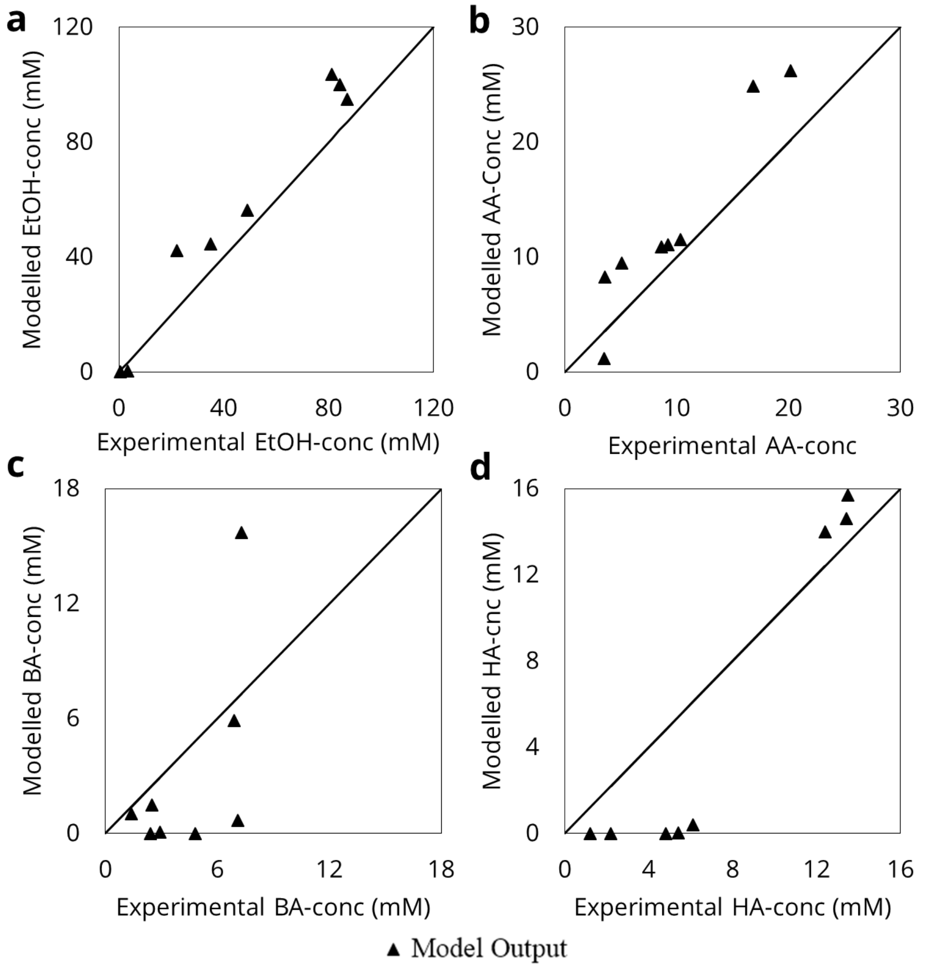

Figure S.10. Comparison of continuous dynamic ODE-model to literature data [3]. Bisector represents outcome of a model perfectly simulating chemostat conditions. Full triangles (▲) show output of chemostat model using experimentally determined parameters in this study. Subfigures a-d show different simulated conditions, cf. Table S.II.

S.2.10. Production kinetics of *Clostridium kluyveri* in 96-WP

To validate the dynamic model for growth of and production by *Clostridium kluyveri* data was collected of a batch growth experiment in 96-WP in the anaerobic closet.

A data quality control was performed by looking at replicability within a plate, as well as replicability between plates. The first shows whether there is any influence due to the disturbances during sampling (i.e. measuring OD, removing lid, sampling, refilling emptied wells and putting back the lid), while the latter shows how replicable the experiment as a whole is.

The replicability within a plate is excellent; average OD of the sampled wells and those wells sampled at the last timepoint were within 1 standard deviation from each other, with only one exception (Figure S.12E at 40h). This implies that the sampling procedure does not disturb the growth of *Clostridium kluyveri*.

Replicability between plates, however, is rather low, as demonstrated by Figure S.13. Figure S.13A shows the case for the High AA-condition, where mainly λ is very variable between experiments. For the Low AA-condition (Figure S.13B) not only λ varies between experiments (especially Plate 1), but also the final OD changes between supposed replicate experiments (Plate 2 at approx. half the OD of Plate 1 and 3). Because of this, the experiment as a whole was not considered for model validation, but for each condition, the – subjectively – best experiment was selected, based on the number of sampling points during the exponential phase; Plate 2 for High AA, Plate 3 for Low AA.

Profiles of product formation and substrate production for both conditions and all plates (Figure S.14) show some patterns across experiments: (1) the accumulation of HA is not necessarily preceded by accumulation of BA in the environment, seemingly contradicting literature data[9], however, the resolution of the data reported here is lower due to the limitation of the number of timepoints; (2) BA appears to have a peak concentration during the

exponential phase, after which concentrations of BA decrease again – in accordance with what is reported in literature[9] and; (3) Production of HA does not stop when growth stops, due to maintenance metabolism in the stationary phase. What causes the switch from exponential growth to stationary phase is not clear, but growth stops before toxic concentrations of HA are reached in the case of High AA. In the case of Low AA, toxic concentrations are never reached, yet growth stops before reaching the highest HA-concentrations and substrate is never completely depleted.

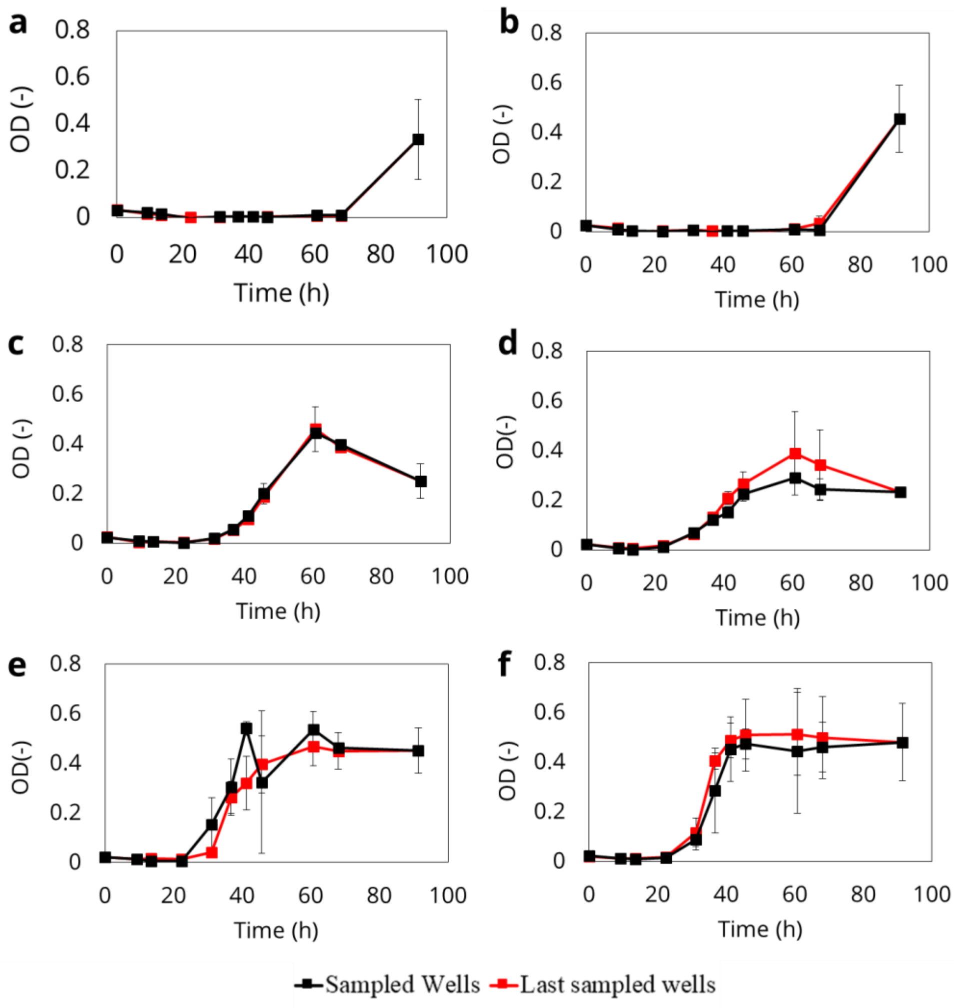

Figure S.12. Comparison within a plate of replicability of growth. Black squares (■) display OD of the wells sampled at each timepoint, red squares (■) represent OD of the wells sampled at the last timepoint (91.3 h) over the course of the experiment. Error bars indicate standard deviation over 3 wells (n=3). Figures A and B High AA and Low AA conditions respectively for Plate 1, C and D show High AA and Low AA conditions respectively for Plate 2, E and F show High AA and Low AA conditions respectively for Plate 3.

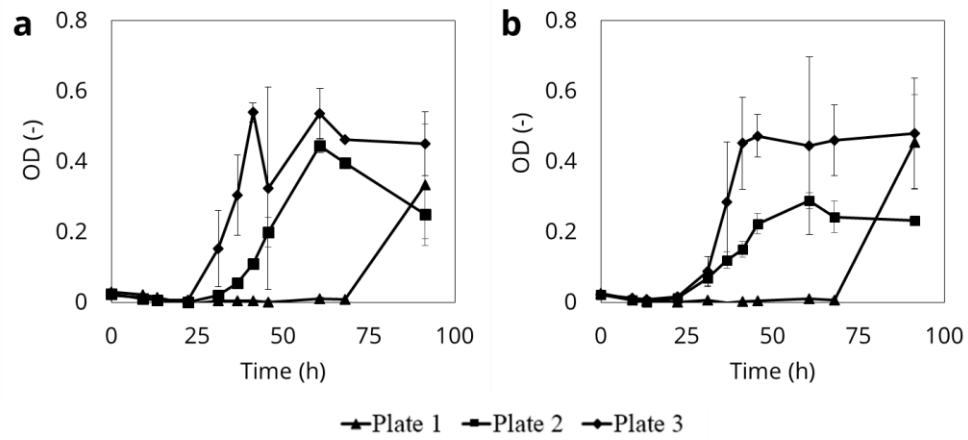

Figure S.13. Comparison of growth between plates. OD of the sampled wells at each timepoint for Plate 1, Plate 2 and Plate 3 are represented by full black triangles (▲), squares (■) and diamonds (◆) respectively. Error bars indicate standard deviation over 3 wells (n=3). A shows results for High AA in each plate, B shows the same for Low AA.

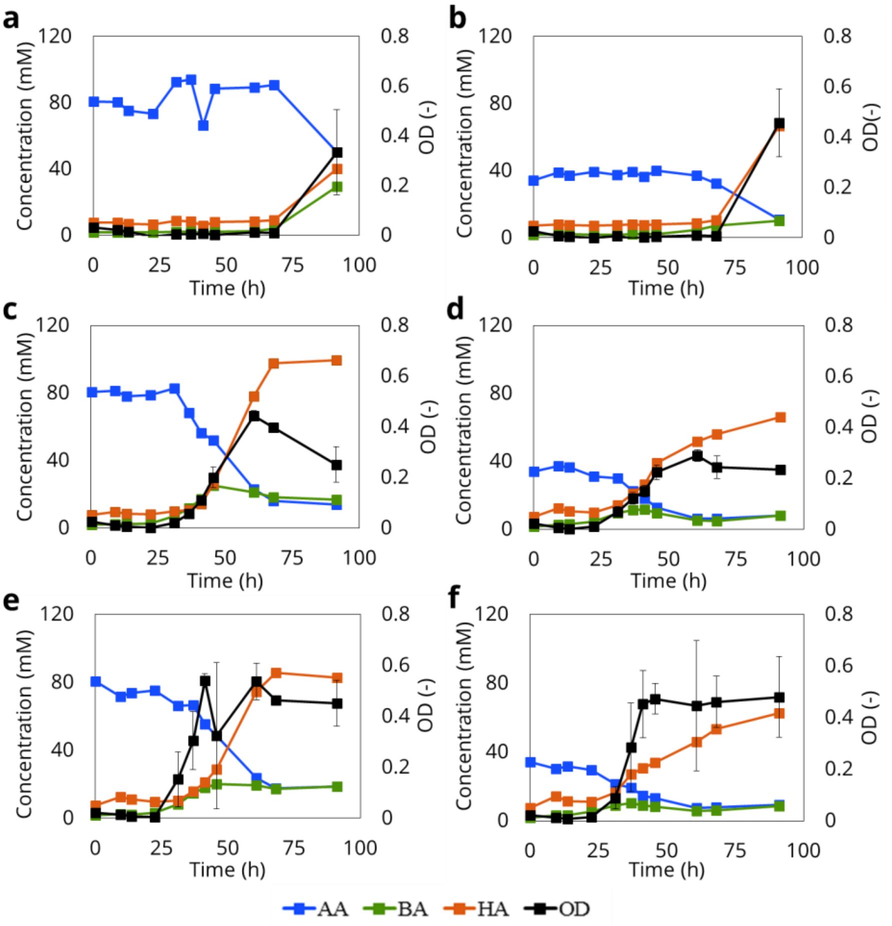

Figure S.14. Kinetics of substrate consumption and product formation by Clostridium kluyveri over the course of the experiment for each plate (a, c and e show High AA for Plate 1, 2 and 3 respectively, b, d and f show Low AA for Plate 1, 2 and 3). Blue(■), brown(■) and green(■) squares show concentrations of AA, BA and HA respectively after pooling of the wells (n=1) on the left vertical axis. Black squares (■) show average OD of wells at time of sampling, error bars represent standard deviation over replicates (n=3).

S.2.11. Assessment of buffering capacity

The medium used in these experiments contained less buffer capacity than the conventional DSM52 medium. In Balch tubes, no CO_2 was present in the headspace for buffering, while the anaerobic closet contained 10% CO_2 instead of the recommended 20% CO_2. It was already shown in the manuscript that final product concentrations in Balch tubes and 96-WP were very similar, giving a first indication *C. kluyveri* was not majorly affected by this change. Secondly, the final pH (Table S.VII), shows a similar trend. pH at the end of the experiment is slightly higher in the anaerobic closet, but always within the pH range for optimum growth of *C. kluyveri*[8]. Additionally, because the growth curve is log transformed before fitting the growth curve, the early exponential phase will carry greater weight in determination of μ. In this phase of growth, conditions are still close to initial conditions, and the buffering capacity is of lower importance.

Table S.VII. Initial pH and final pH for incubations in Balch tubes and 96-WP in experiment G, varying the initial HA concentration between 0 and 184m mM HA. Above a concentration of 90 mM HA no more growth occured, and differences in pH are solely physicochemical in nature

Initial HA (mM)	Initial pH	Final pH Balch tubes	Final pH 96-WP
5	7.79	6.45	7.04
17	7.87	6.54	6.98
27	7.9	6.72	6.95
39	7.81	6.62	6.94
52	7.91	7.04	7.24
61	7.89	7.99	7.88
75	7.85	7.8	7.96
89	7.42	7.9	8.09
95	7.8	8.05	8.07
105	7.51	7.89	8.25
113	7.82	7.93	8.22
128	7.81	7.99	8.19
140	7.83	8.15	8.19
145	7.83	8.12	8.21
151	7.85	8.03	8.21
160	7.88	8.29	8.23
184	7.77	8.18	8.18
Abiotic control	7.61	7.92	8.43
No substrate control	8.24	8.29	8.25
Salinity control	7.76	6.32	7.28

S.3. Discussion

S.3.1. Thermodynamical impact of H_2 accumulation

H_2 is a product of chain elongation by *C. kluyveri*, so increasing H_2 partial pressures in the gas headspace decreases the energy gain (ΔG^0) from the chain elongation metabolism. To assess whether H_2 accumulation in Balch tubes could hinder the energy generation in this metabolism, theoretical calculations were performed, tracking ΔG^1 during reaction, with vs. without H_2 accumulation. This was done for the hypothetical situation where only the first step of the chain elongation process proceeds (i.e. EtOH and AA to BA), as well as the situation where both steps of the metabolism occur in series, and only HA is being produced and no BA (see S.1.7.). This was compared to the experimentally observed endpoint of the reaction.

For both cases, a clear impact on ΔG^1 of the accumulation of H_2 can be observed, causing the case with accumulation of H_2 in the headspace to cross the maximum feasible ΔG^1, and thus become thermodynamically unfeasible (Figure S.13.). Some of the assumptions made for the calculations (see S.1.7.) may however be imperfect: (i) Formation of longer chain alcohols has been observed by *C. kluyveri*[10], implying at least some H_2 lost in the reduction of carboxylic acids to alcohols. (ii) Based on the determined Y_{EtOH}, 6±1.7% of the EtOH consumed ends up in the form of biomass, again implying a reduction of H_2 production. (iii) Metabolic studies have shown that *C. kluyveri* uses a slightly alternative pathway under low substrate, thermodynamically limiting conditions. This alternative pathway generates less ATP per reaction step (i.e. lower kinetics), but can take place at higher ΔG^1, and could be used as a means to continue growing under high H_2 partial pressures[5]. If the ΔG^1-limit is a switch between metabolisms, rather than an endpoint, then H_2 partial pressure is approx. 1.2 atm and approx. 73 mM of HA would be produced at the time of the switch, which is already close to the stationary phase of the growth curve (Figure S.12.). Because these conditions fall outside the scope of the study, they were not included in the kinetic model. If the assumption of thermodynamics forcing a switch between high and low energy yielding metabolism shows true, another set of kinetics could be determined at high H_2 partial pressures, and included in the kinetic model.

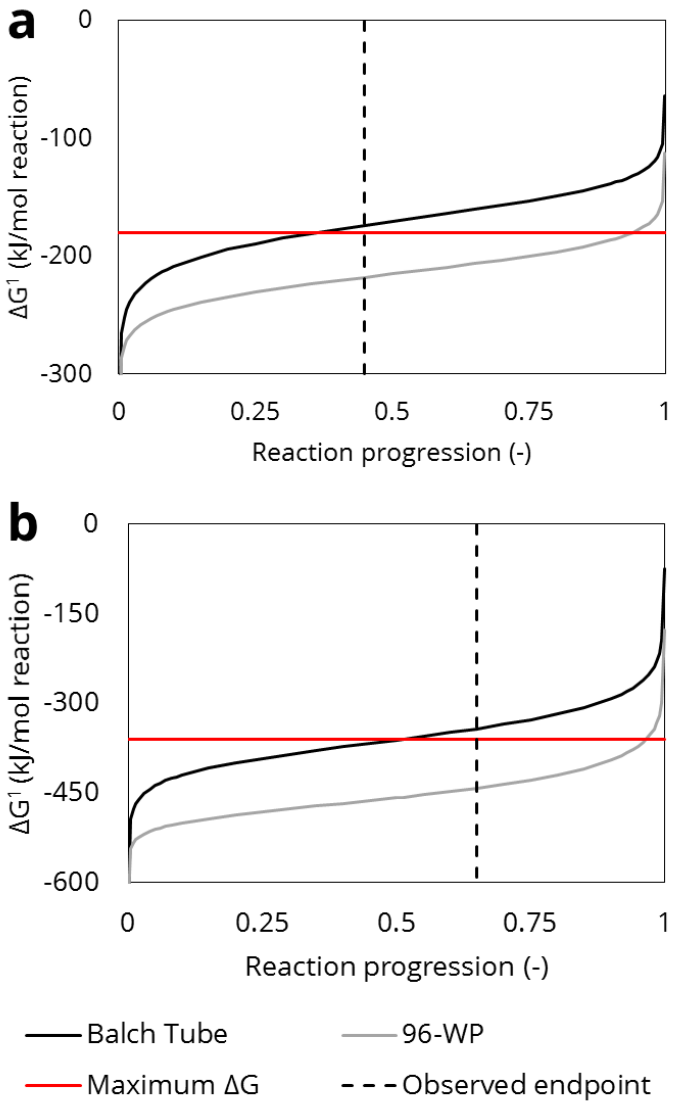

Figure S.15. Gibbs free energy (ΔG^1) as a function of reaction progression. ΔG^0_r was calculated from reference values[4] and was corrected for temperature (37°C or 310.15 K), and pH (pH = 7). Starting in DSM 52 medium as initial conditions, A shows the evolution of ΔG^1 if only the first step of the chain elongation reaction takes place as a function of reaction progression (i.e. fraction of AA consumed). B shows the same if both reaction steps occur - only HA is a carboxylate product – as a function of reaction progression (i.e. fraction of EtOH consumed). Black line shows the case with accumulation of H_2 in a Balch Tube, while the grey line shows evolution of ΔG^1 in case no H_2 is accumulated, cf. 96-WP. Red line indicates the maximum ΔG^1 value generated in the chain elongation steps to generate sufficient ATP at 72 kJ.mol ATP^{-1} [5]. The dashed black line indicates where reaction has been observed to stop, based on H_2 partial pressure (Fig A) or HA-concentration (Fig B).

S.3. Bibliography

1. R Core Team. R: A language and environment for statistical computing. (2015).
2. Van Nevel, S., Koetzsch, S., Weilenmann, H. U., Boon, N. & Hammes, F. Routine bacterial analysis with automated flow cytometry. *J. Microbiol. Methods* **94,** 73–76 (2013).
3. Kenealy, W. R. & Waselefsky, D. M. Studies on the substrate range of Clostridium kluyveri; the use of propanol and succinate. *Arch. Microbiol.* **141,** 187–194 (1985).
4. Kleerebezem, R. & Van Loosdrecht, M. C. M. A generalized method for thermodynamic state analysis of environmental systems. *Crit. Rev. Environ. Sci. Technol.* **40,** 1–54 (2010).
5. Angenent, L. T. *et al.* Chain Elongation with Reactor Microbiomes : Open-Culture Biotechnology To Produce Biochemicals. *Environ. Sci. Technol.* **50,** 2796–2810 (2016).
6. Barker, H. A. & Taha, M. Clostridium Kluyverii, an organism concerned in the formation of caproic acid from ethyl alcohol. *J. Bacteriol.* **43,** 347–363 (1941).
7. Smith, G. M., Kim, B. W., Franke, A. A. & Roberts, J. D. 13C NMR Studies of Butyric Fermentation in Clostridium kluyveri. *J. Biol. Chem.* **260,** 13509–13512 (1985).
8. Gildemyn, S. *et al.* Upgrading syngas fermentation effluent using Clostridium kluyveri in a continuous fermentation. *Biotechnol. Biofuels* **10,** 83 (2017).
9. Thauer, R. K., Jungermann, K., Henninger, H., Wenning, J. & Decker, K. The Energy Metabolism of Clostridium kluyveri. *Eur. J. Biochem.* **4,** 173–180 (1968).
10. Seedorf, H. *et al.* The genome of Clostridium kluyveri , a strict anaerobe. *Proc. Natl. Acad. Sci. U. S. A.* **105,** 2128–2133 (2008).
